# Genozip: a universal extensible genomic data compressor

**DOI:** 10.1093/bioinformatics/btab102

**Published:** 2021-02-15

**Authors:** Divon Lan, Ray Tobler, Yassine Souilmi, Bastien Llamas

**Affiliations:** Australian Centre for Ancient DNA, School of Biological Sciences, The Environment Institute, Faculty of Sciences, The University of Adelaide, Adelaide, SA 5005, Australia; Australian Centre for Ancient DNA, School of Biological Sciences, The Environment Institute, Faculty of Sciences, The University of Adelaide, Adelaide, SA 5005, Australia; Centre of Excellence for Australian Biodiversity and Heritage (CABAH), School of Biological Sciences, University of Adelaide, Adelaide, SA 5005, Australia; Australian Centre for Ancient DNA, School of Biological Sciences, The Environment Institute, Faculty of Sciences, The University of Adelaide, Adelaide, SA 5005, Australia; National Centre for Indigenous Genomics, Australian National University, Canberra, ACT 0200, Australia; Australian Centre for Ancient DNA, School of Biological Sciences, The Environment Institute, Faculty of Sciences, The University of Adelaide, Adelaide, SA 5005, Australia; Centre of Excellence for Australian Biodiversity and Heritage (CABAH), School of Biological Sciences, University of Adelaide, Adelaide, SA 5005, Australia; National Centre for Indigenous Genomics, Australian National University, Canberra, ACT 0200, Australia

## Abstract

We present Genozip, a universal and fully featured compression software for genomic data. Genozip is designed to be a general-purpose software and a development framework for genomic compression by providing five core capabilities—universality (support for all common genomic file formats), high compression ratios, speed, feature-richness and extensibility. Genozip delivers high-performance compression for widelyused genomic data formats in genomics research, namely FASTQ, SAM/BAM/CRAM, VCF, GVF, FASTA, PHYLIP and 23andMe formats. Our test results show that Genozip is fast and achieves greatly improved compression ratios, even when the files are already compressed. Further, Genozip is architected with a separation of the Genozip *Framework* from file-format-specific *Segmenters* and data-type-specific *Codecs*. With this, we intend for Genozip to be a general-purpose compression platform where researchers can implement compression for additional file formats, as well as new codecs for data types or fields within files, in the future. We anticipate that this will ultimately increase the visibility and adoption of these algorithms by the user community, thereby accelerating further innovation in this space.

**Availability and implementation:**

Genozip is written in C. The code is open-source and available on http://www.genozip.com. The package is free for non-commercial use. It is distributed through the Conda package manager, github, and as a Docker container on DockerHub. Genozip is tested on Linux, Mac and Windows.

**Supplementary information:**

Supplementary data are available at *Bioinformatics* online.

## 1 Introduction

Genomic data production is growing rapidly as sequencing prices continue to drop, making data storage and transfer a core issue for researchers, healthcare providers, service facilities and private companies. To date, most users have relied upon compression software that implements the RFC 1951 format [Deutsch, 1996; e.g.gzip, bgzip (Li, 2011) and others], a general-purpose compression format that was designed decades ago and is not specifically tailored for genomic data.

Many novel algorithms have emerged in recent years that effectively compress one or more of the data types embedded in genomic files [e.g.GTShark (Deorowicz and Danek, 2019) and SPRING (Chandak et al., 2019)]. However, these algorithms are typically implemented within a rudimentary software package that inadvertently lacks the breadth of features required for a software to be useful in many real-world use cases; most importantly, most work with only one of the common file formats. These limitations have meant that none of these software packages are currently widely used by the genomic researcher and practitioner community.

Here, we introduce a new version of the compression software Genozip, which has been nearly completely re-written from an earlier version designed to compress VCF files (Lan et al., 2020). Genozip now offers five core capabilities:

1. Universality—Genozip supports all common genomic file formats—FASTQ, SAM/BAM/CRAM, VCF, GVF, FASTA, PHYLIP and 23andMe.
2. High compression ratios—better than all other universal tools tested.
3. Speed—in most cases, faster than other tools.
4. Feature richness—providing an array of features that allow integration into pipelines, specification of compression options and development tools to allow developers to extend Genozip easily.
5. Extensibility—with a clear separation of the Genozip Framework from the file formats being compressed and from the codecs used for compression, it is fairly easy to add support for more file formats as well as new codecs to improve compression of specific data types of any specific fields within genomic files.

## 2 Software description

Genozip provides a command line interface that consists of four commands: genozip for compression, genounzip for decompression, genocat to display or subset a compressed file, and genols to show metadata associated with the compressed files.

Genozip is currently optimized to compress FASTQ, FASTA, SAM/BAM/CRAM, VCF/BCF, GVF, PHYLIP and 23andMe files, including files that are already compressed into .*gz*, .*bz2*or .*xz* formats. However, Genozip can also compress any other file format. Compression of .cram, .bcf or .xz files requires the software packages samtools, bcftools or xz, respectively, to be available in the PATH environment variable. Genozip allows multiple files of identical or different formats to be specified in the command line. Files that share a common format can be *bound* together with genozip –output and subsequently unbound with genounzip –unbind. This functionality is beneficial for packaging a large number of samples together for delivery or archiving.

Genozip can be integrated into analytical pipelines in two ways. First, genozip and genounzip may be used with pipes. Second, genocat provides random-access to user-specified sections of a .genozip file and facilitates file subsetting. When using genocat to subset files, the targeted data are identified using the –samples option for VCF files and the –regions option for SAM, VCF, FASTA, GVF and 23andMe file types. –downsampledownsamples any file type. Further, because .genozip files are indexed during data compression, a separate indexing step is not required.

In addition, genocat offers built-in file format *translation*, and currently offers *translations* between SAM and BAM, from SAM or BAM to FASTQ, between FASTA and PHYLIP and from 23andMe to VCF, using genounzip’s–bam, –sam, –fastq, –phylip, –fasta and –vcf options, respectively.

Genozip offers a range of data integrity and security options. To support data security requirements that comply with ethical standards now expected for modern genomic projects, Genozip allows encryption of the data using the –password option. With this option, data are encrypted with the standard Advanced Encryption Standard (AES) algorithm (Fips, 2009), using the strongest mode available (256 bits). To ensure data integrity, Genozip includes a built-in MD5 (Rivest, 1992) option triggered by using –md5 or –test. This calculates (in genozip) or verifies (in genounzip and genocat) the MD5 sum of the source data on the fly and stores it within the compressed genozip file. This MD5 sum is then viewable using genols.

Genozip offers two lossless compression modes: –best, which is the default and results in the highest compression ratio, and –fast, which optimizes compression speed at the cost of somewhat reduced compression ratios (see Supplementary Section S12). While Genozip is strictly lossless by default, a lossy–optimise (or –optimize) option is also offered, which further improves compression by modifying the data in ways that typically do not impact downstream analysis (See Supplementary Section S3).

In addition, Genozip supports compression with or without a reference genome sequence. Providing a reference improves compression of the sequence data component in SAM/BAM/CRAM, FASTQ and VCF files. A reference file may be generated from a FASTA file with genozip –make-reference and used with genozip –reference or –REFERENCE. The latter option stores information from the reference within the resulting compressed file, obviating the need to provide the reference as a separate file during the decompression step. Including the reference information within the compressed file is particularly useful when binding several genomic data files together for delivery.

Finally, fine level information on various aspects of the data compression can be accessed by the user via the large suite of –show options (see Supplementary Section S8). For instance, –show-stats provides compression statistics broken down by data type within the file. We anticipate that such information will be insightful for end-users and particularly useful when developing new compression algorithms.

## 3 Materials and methods

### 3.1 Framework and architecture

**Fig. 1.**
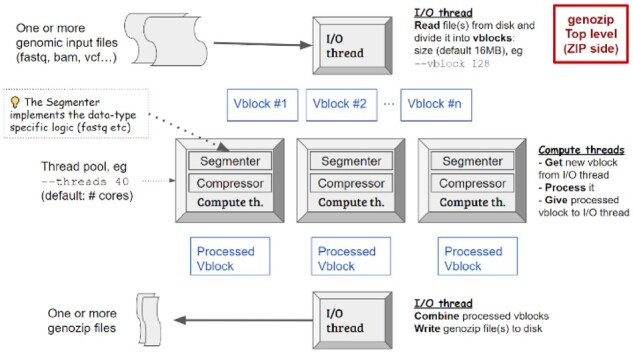
Genozip high-level architecture. The Genozip framework interprets and reads the input file(s) in the main thread (I/O thread) and divides them into vblocks, which are then segmented. Segmentation is followed by the compression step. Compressed vblocks are sent back to the I/O thread to create the.genozip output(s)

The Genozip framework (Fig. 1) interprets the user’s command line, reads the source genomic file (referred to as the *txt file*) and divides it into *vblocks*. Each vblock comprises a certain number of full *txt file* lines, limited by size that is determined by the user with the –vblock option (default: 16MB). By default, a *line* means an actual ASCII line in the *txt file*; however, this is flexible—e.g. for FASTQ, a *line* comprises four textual lines and for BAM it comprises one alignment record.

Once the Genozip framework has read the *vblock txt data* into memory using its main thread (called the *I/O thread*; Fig. 1), a separate *compute thread* is spawned to segment the *vblock*. This segmentation step is followed by the final compression step that ultimately generates *z data*, which is the final compressed data for the *vblock*. When the compression step is completed, the compute thread terminates and the compressed *vblock*is handed back to the *I/O thread* that appends it to the .genozip compressed file being generated on disk.

### 3.2. The segmentation step

A *segmenter*is a module that is specific to the file format being compressed. Genozip currently has nine segmenters, one each for FASTQ, FASTA, SAM, BAM, VCF, GVF, PHYLIP, 23andMe and Generic. If samtools (Li *et al.*, 2009) is also installed, the SAM segmenter can also handle CRAM files by reading them as SAM. The *Generic* segmenter handles all other file formats for which genozip does not have a *segmenter* in a default manner. Importantly, interested parties can add more segmenters to Genozip in the future.

The *segmenter*is called by the Genozip framework to work on one line of *txt data* at a time, and the job of the *segmenter*is to segment this line into its individual data components, store these in *contexts* (which are described in detail in Supplementary Section S2) and declare how each context should be handled in the compression stage.

The segmenter starts by breaking up the *txt line* into the top-level data fields and deciding what to do with each data field. Broadly, it has six options:

1. Placing the data directly in its appropriate  *context*. We refer to a short string of data inserted into a context as a *snip*. Each new *snip* encountered by the Genozip framework is added to a *dictionary* within each *context*, and an index is added to the *dictionary* entry in a data buffer for this *context* called the *b250 buffer.* Accordingly, the .genozip file stores each *snip* only once and uses a numeric index to point to it throughout the file.
2. Further segmenting a field into its subfields: Rather than making a *snip* of the entire field data as it appears in the file, the segmenter can insert a special *snip* type called a *Container*, which defines the structure of the data of this field, where the data itself is stored in other *contexts* that are named in the container. *Containers* can define records containing multiple types of data, as well as arrays of similar data elements or arrays of records. The entire *vblock* is described as a single *Container snip* placed in the TOPLEVEL *context*. This is a key feature that enables the decompressor to be generic. Indeed, in most cases, the decompressor need not have any built-in awareness of the details of each file format. The file format structure is encoded in the data itself, and a *vblock* may be reconstructed by traversing the data starting from the TOPLEVEL.
3. This is a key feature that enables the decompressor to be generic. Indeed, in most cases, the decompressor need not have any built-in awareness of the details of each file format. The file format structure is encoded in the data itself, and a *vblock* may be reconstructed by traversing the data starting from the TOPLEVEL.

1. Exploiting known relationships between fields, subsequent lines  and/or external data to improve the compression. For that, the segmenter may define *contexts* as needed—for example, it may store multiple fields in a single context or may decompose a field into multiple contexts. It can be as simple as exploiting a mathematical relationship between fields, but it can also be complex—for example, the sequence data in FASTQ and SAM are aligned to a reference if the –reference option is used.
2. Using one of the Genozip’s framework built-in algorithms. Some relationships occur frequently, for which Genozip has built-in algorithms. These include the *seg_pos* algorithm that exploits the nearness of position data in subsequent lines, if it exists and *seg_id* algorithm that handles ID data that starts with an alphabetical prefix followed by a number (such as ‘rs23424’) as well as LOOKUP and DELTA versus another field on the same line or versus the same field in a previous line or versus the pair file (in case of paired-end FASTQ files). Details about these built-in algorithms can be found in Supplementary Section S2.
3. Preparing the data for a  *specific*  codec. Rather than inserting a *snip*, the segmenter can store the data of a field in the *local* buffer of the *context* in any proprietary way, in preparation for consumption by a *specific* codec in the compression stage.
4. Declaring a  *context*  to be an  *alias.* There are cases where multiple fields contain data with similar characteristics, in which case storing them in a single *context* can improve compression. To achieve this, we can define a *context* as an *alias* of another, essentially sharing their data. For example, in SAM format, there are multiple Optional tags that express data in CIGAR format (MC:Z, OC:Z and others), which are all defined as aliases of a *context* named @CIGAR.

In the *Generic*segmenter used for unrecognized file formats, the segmenter is trivial and does not actually segment the data—instead, the entire *vblock* data is placed in a *local* buffer of a single context.

A detailed example of how these six options work is in Supplementary Section S2, as well as a full list of how each of the nine segmenters in Genozip handles each data field.

### 3.3. Context management

Segmentation step: Each *vblock* maintains its own set of *contexts—*the set consisting of one *context* for each data component. A context is a data structure that includes the *dictionary*, *b250*, and *local* data buffers as well as additional information.

Context merging step: We maintain one global set of similar *contexts* within an object called the *z_file* to which we merge vblock*contexts’* dictionary data after the segmentation is completed for a *vblock*, thereby incrementally creating a global dictionary containing, in a particular *z_datacontext*, all values of that appear for that data component in the entire file (except for singletons—see Supplementary Section S2).

Cloning step: When a new *vblock* is created, the current dictionary and related information of each *context* are *cloned* from the *z_file* to the new *vblock* by the framework.

Writing step: After the compute thread terminates and the *vblock* is handed back to the I/O thread, the I/O thread writes the *vblock’*s*z_data* (containing *b250 and local* sections) to the output .genozip file. The merged dictionary data is written upon the completion of computing of all *vblocks*.

*Context* cloning, concurrent *dictionary* access and *context* merging in a multi-threaded environment are difficult, even more so with minimal synchronization between threads to avoid a bottleneck that would limit scaling CPU cores. We employ advanced multi-threading mechanisms that ensure that all threads can operate on the same dictionaries concurrently while minimizing the use of synchronization objects like mutexes, minimizing memory copies, and ensuring O(1) dictionary lookups, uniqueness of dictionary entries and thread-safety. Details of how this is done are in Supplementary Section S6.

### 3.4 The compression step

Within the *compute thread* of any specific *vblock*, and once the segmentation is complete for all lines and the contexts dictionaries have been merged back into *z_file*, the framework proceeds to compress the two buffers of each *context* present in this vblock—namely, the *b250* and the *local* buffers. Each buffer is compressed with one of the available codecs. There are two types of codecs in Genozip:

*Generic* codecs—these are lzma (Pavlov, 2007), bz2 (Seward, 1996), bsc (http://libbsc.com/) and *none*. The first three are standard codecs for which Genozip utilizes a modified version of the standard libraries, and the fourth is a codec that essentially keeps the data as-is.

*Specific* codecs—these are additional codecs that compress a specific data type better than the generic codecs and would often be *complex* codecs—which means that they will perform some processing of the data, and then complete the compression by applying one or more of the built-in codecs. *Specific* codecs can be added to compress any specific field of any genomic file format.

For the *b250* and *local* buffers of each *context*, the codec is selected automatically by sampling approximately 100KB of the buffer data in the first *vblock* in which this *context* is first encountered and compressing it with each of the four codecs. The best codec is selected by an algorithm that chooses the codec with the best compression ratio unless the compression ratio between the best two codecs is close enough, and the execution time is different enough, in which case it selects the faster codec of the two. Subsequent *vblocks* use the same codec and need not test again. In –fast mode, a modified selection algorithm is used that is biased towards speed even at the expense of a small difference in compression.

A segmenter may specify a codec for the *local* buffer of any particular *context*, overriding the automatic selection. In the segmenters provided, we use this privilege only when we set the codec to a *specific* codec.

Genozip currently has four *specific* codecs:

1. *acgt—*used for compression of a sequence of nucleotides, which is expected to contain mostly, but not necessarily exclusively, ‘A’, ‘C’, ‘G’ or ‘T’ characters. It is used to compress FASTA sequence data and characters (bases) from the SEQ field of FASTQ and SAM file formats that are not mapped to a reference.
2. *domqual—*used for compression of a string of Phred quality-scores in SAM and FASTQ formats, in the common case where there is one dominant score value.
3. *hapmat—*used for compression of a matrix of haplotypes derived from FORMAT/GT fields in VCF. The algorithm is described in (Lan*et al.*, 2020) and has been re-implemented to serve as a codec.
4. *gtshark—*triggered by the –gtshark option, utilizes the software package GTShark (Deorowicz and Danek, 2019) as a codec for the same haplotype matrix as *hapmat* as an alternative to *hapmat*. This was already implemented in (Lan*et al.*, 2020), where we have shown it to be significantly better but significantly slower than *hapmat* for the FORMAT/GT data component in VCF files that have a large number of samples. It has been re-implemented as a codec for FORMAT/GT on top of the new framework and with a new fast in-memory (rather than disk-based) communication channel between genozip and gtshark. This is an example of how Genozip can be easily extended to incorporate new codecs for specific data types.

More details on the algorithms for each of these codecs can be found in Supplementary Section S6.

### 3.5 Compressing against a reference

Genozip does not require a reference but takes advantage if one is available to better compress FASTQ, SAM/BAM and VCF data.

To use a reference with Genozip, a *Genozip reference file* must first be created using genozip–make-reference. This is a one-time step for any particular reference FASTA file. The Genozip reference file creation is implemented by segmenting the reference FASTA data with a specialized segmenter, which generates a Genozip file containing a pre-processed version of the reference data in a format that is readily usable by Genozip, as well as hash tables for use of the Genozip Aligner, indexing data and additional metadata.

When using a particular Genozip reference file to compress data for the first time, Genozip generates two cache files. These files are used to accelerate the loading of the reference data and the Genozip Aligner hash tables in subsequent executions of Genozip and may be deleted if such acceleration is not needed. The acceleration is achieved by loading the cache files, if they exist, using the operating system’s paging system rather than libc allocated memory, allowing portions of the reference data to be paged-in as needed, and also enables sharing of the loaded pages between concurrently running Genozip processes, resulting in reduced memory consumption and instantaneous loading in the case of concurrent Genozip instances.

The VCF segmenter uses reference data to avoid storing REF and/or ALT data and referring to the reference if possible. Since the REF and ALT fields usually represent only a small fraction of the information content of a VCF file, the gains are modest, however.

The SAM and BAM segmenters use reference data in two different ways, depending on whether the *txt line* being segmented is aligned (i.e. contains values in the RNAME, POS and CIGAR fields) or not, and the FASTQ segmenter uses the reference similar unaligned SAM/BAM:

1. For an aligned SAM/BAM/CRAM *txt line*, the segmenter decomposes the data into three *contexts*: SQBITMAP, NONREF and NONREF_X. SQBITMAP is a bitmap consisting of a bit for every base in the sequence that ‘consumes a reference’, as defined in the SAM specification (https://samtools.github.io/hts-specs/SAMv1.pdfpage 8) according to the CIGAR string. The bit is set to 1 if the base is the same as the base in the reference data at its position. If not, the bit is set to 0, and the base character is placed in NONREF. Bases in the sequence that ‘do not consume a reference’, according to the CIGAR string, are also placed in NONREF. NONREF is set to be compressed with the *acgt* codec that requires a second *context* for the CODEC_XCGT data, which is NONREF_X (see Supplementary Section S6).
2. For an unaligned SAM/BAM/CRAM *txt line* and a FASTQ sequence line, the Genozip Aligner is used. It utilizes the same three *contexts* described above and two additional ones: GPOS and STRAND. The Aligner algorithm (see Supplementary Section S4) finds the position in the reference to which the sequence string at hand best aligns. This algorithm is extremely fast as it does not attempt to find the biologically correct alignment, just one that compresses well. The aligner determines the location in the reference, using a coordinate called *gpos*(Global Position) - which is a single 32-bit unsigned integer covering the entire reference genome, and indicates whether it is forward or reverse complement relative to the sequence (which we call *strand*). The segmenter then stores the *gpos* and *strand* in the *local* buffers of the GPOS and STRAND contexts, respectively (the strand is stored as a bitmap with ‘1’ meaning forward) and proceeds to populate the SQBITMAP and NONREF contexts as before, based on whether or not each base in the sequence matches the corresponding base in the forward or reverse complement reference.

### 3.6 Indexing

While Genozip is designed as a compression tool rather than a data analysis tool, it also contains some capabilities that allow direct integration into analysis pipelines. Chief among these, is indexing of the data done by the Genozip framework during segmentation, which then allows subsetting the data with the genocat –regions option: a segmenter may notify the Genozip framework of the chromosome (or contig) and position of each line being segmented. As the segmentation progresses, the framework collects data per vblock—namely, it records which chromosomes appear in the vblock, and the minimum and maximum position of each chromosome within the vblock. These data are then emitted to the generated compressed genozip file as the SEC_RANDOM_ACCESS section.

During genocat–regions,vblocks that contain no data from the requested regions are skipped entirely, while vblocks that do contain data from the requested regions are decompressed, but only lines that are included in the requested regions are emitted.

In addition, Genozip reference files are also indexed in the same way, so when subsetting a file that requires a reference (i.e. the –reference option is used), Genozip only reads the vblocks of the reference file that overlap with the regions requested.

Currently, the segmenters for VCF, SAM, BAM, GVF and 23andMe implement this capability.

This indexing method is more coarse-grained than the BGZF-block level indexing that is common in standard indexes of genomic file formats, as subsetting requires decompression of entire vblocks (16MB of txt data in the default configuration) versus just BGZF blocks (64KB of data), and hence subsetting is significantly slower. However, in practice, this may be sufficient for many analysis applications.

## 4 Results

We evaluated the performance of Genozip by compressing genomic files as they most commonly appear in real-world research and clinical situations—namely, already compressed in fastq.gz, BAM, CRAM and vcf.gz formats. Regarding CRAM, we tested two different commonly used versions of CRAM files—a version containing the same data as the BAM file and a version optimizedby binning quality data. For VCF, we tested a single-sample file. We previously reported the compression performance of multi-sample VCF using an earlier version of the HapMat codec in Lan*et al.* (2020). For BAM, CRAM and FASTQ, we also tested with Genozip’s–optimise option.

The FASTQ, BAM and VCF files (Table 1 with further details in Supplementary Table S10) were obtained from a public FTP server of the National Center for Biotechnology Information (NCBI), while the CRAM files were generated from the BAM file usingScramble (Bonfield, 2014) with the highest compression ratio (-9 option) and, in addition, for the binned-quality CRAM, with the quality-binning option -B (Table 1). The reference file used was based on a modified version of GRCh37 as required by the particular BAM file tested (see Supplementary Section S12) and was prepared with: genozip –make-reference $grch37-fasta-file.

**Table 1.** Files used for testing against already-compressed files

File type	File size	**Genozip command** —optimise added for the Optimised test
.fastq.gz	3.6 GB (R1+R2)	genozip—pair $file-R1 $file-R2 -e $ref-file
.bam	147 GB	genozip $file -e $ref-file
.cram (lossless)	102 GB	genozip $file -e $ref-file
.cram (binned)	79.5 GB	genozip $file -e $ref-file
.vcf.gz	128 MB	genozip $file -e $ref-file

*Note*: See more details in Supplementary Table S10.

**Fig. 2.**
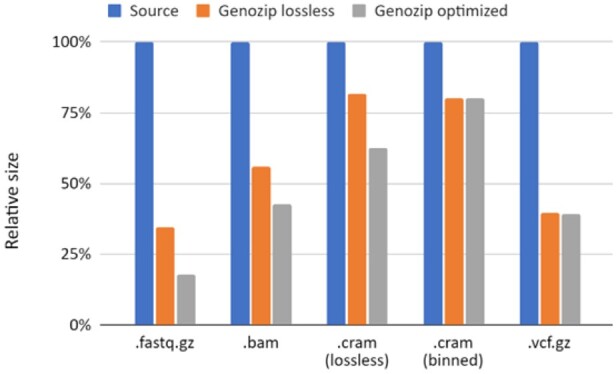
Sizes of Genozip-compressed files relative to already-compressed source files.The blue bars represent the source files (see Table 1), with the corresponding file extensions at the bottom. The orange and grey bars are for Genozip compression with the default, lossless mode and the –optimise option, respectively. See also results in Supplementary Table S11

Genozip improved the compression of these already-compressed files in every scenario we tested by a 1.2–5.7 factor (Fig. 2 as well as Supplementary Table S11 in Supplementary Information).

**Fig. 3.**
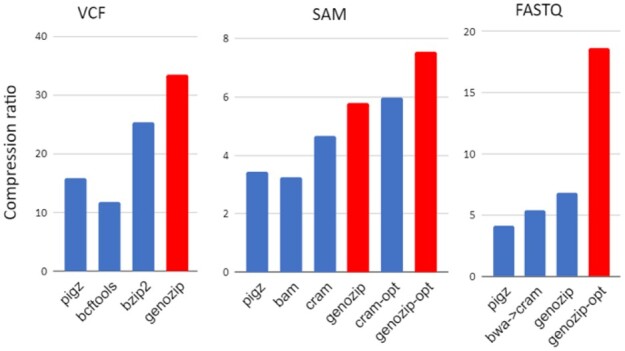
Raw (uncompressed) files benchmark results.The three panels show compression ratios of various relevant compression formats indicated at the bottom relative to uncompressed VCF (left), SAM (middle) and FASTQ (right) files relative. See Supplementary Section S12 for more details

In addition, we performed tests comparing Genozip’s compression ratio on raw (uncompressed) files (Supplementary Table S8 in Supplementary Information), as well as compression and decompression time, to several popular tools. These additional results can be found in Supplementary Section S12 and illustrated in Figure 3, Table 2 and Supplementary Tables S8 and S9. Again, in all cases tested, Genozip outperformed other software for compression ratio by a 1.3-4.4 factor, while also faster than other tools in most, but not all, cases.

**Table 2.** Raw-file benchmark results

Tool	Ratio	Compress time	Decompress time
**VCF**			
Pigz	15.9	1.9 sec	3.1 sec
bcftools	11.7	23.82 sec	21.02 sec
bzip2	25.3	260.05 sec	43.37 sec
genozip	33.6	7.1 sec	6.53 sec
**SAM**			
Pigz	3.4	00:12:40.3	00:34:17.4
Bam	3.2	00:23:16.7	00:29:48.5
Cram	4.7	00:27:58.4	00:17:34.4
genozip	5.8	00:33:41.1	00:27:55.3
cram opt (binned quality)	6.0	00:48:56.1	00:19:10.4
genozip opt (—optimise)	7.6	00:30:51.1	00:20:38.0
**FASTQ**			
Pigz	4.2	00:14:34.5	00:34:17.4
bwa -> cram	5.4	03:42:54.0	00:48:24.7
genozip	6.8	00:16:40.1	00:08:31.7
genozip opt	18.6	00:08:52.3	00:05:26.4

*Note*: Seemore details in Supplementary Table S9.

## 5 Conclusion

Genozip provides not only excellent compression for raw (uncompressed) genomic files, but also provides excellent compression when applied directly to already-compressed genomic files, as is common in real-world applications. Genozip is also universal and works on all common genomic files, uniquely so amongst currently available genomic file compressors.

Further, by providing a modular and extensible architecture, Genozip is also a framework that can be used for rapid development and deployment of new compression algorithms for established or emerging genomic data types and file formats.

## Supplementary Material

btab102_Supplementary_Datay
