# Supplementary material for: Genozip: a universal extensible genomic data compressor
Source: Bioinformatics. 2021 Feb 15;37(16):2225–30. doi: 10.1093/bioinformatics/btab102 (PMC8388020; doi:10.1093/bioinformatics/btab102)
Supplement: btab102_Supplementary_Datay [file btab102_supplementary_datay.pdf]

Genozip - A Universal Extensible Genomic Data Compressor

Supplementary Information

Divon Lan[1,*], Ray Tobler[1,2], Yassine Souilmi[1,3,†,*], Bastien Llamas[1,2,3,†,*]

[1] Australian Centre for Ancient DNA, School of Biological Sciences, The Environment Institute, Faculty of Sciences, The University of Adelaide, Adelaide SA 5005, Australia
[2] Centre of Excellence for Australian Biodiversity and Heritage (CABAH), School of Biological Sciences, University of Adelaide, Adelaide, SA 5005, Australia
[3] National Centre for Indigenous Genomics, Australian National University, Canberra, ACT 0200, Australia

[†] Equal contribution
* Corresponding authors: DL (divon.lan@adelaide.edu.au) and BL (bastien.llamas@adelaide.edu.au)

TABLE OF CONTENTS

1. Genozip high level architecture 3

2. The Segmenter 5
General 5
The segmentation process from the Segmenter’s viewpoint 5
Pre-segmentation Cloning and post-segmentation Merging 8
Singleton detection 9
Hash tables & the Snip Diversity Estimation Algorithm 9
The Snip format 10
The Context data structure 11
The built-in POS algorithm 12
The built-in ID algorithm 13
The built-in Container algorithm 14
The built-in Compound Field algorithm 15
The built-in Special Snip mechanism 16
Full list of contexts used, by file type 17

3. Optimisations 24

4. Compression against a reference and the Genozip Aligner 25
Overview 25
The REFERENCE data 26
The REF_HASH data 27
Compressing aligned SAM data 28
Compressing VCF data (REF and ALT fields) with a reference 30
Compression of FASTQ sequence data and SEQ fields in unaligned lines in SAM data 31
Alternative contig names 34
Discussion 34

5. Compression of FASTQ paired end read files 36

6. Specific codecs 37
acgt: A specific codec for compression of nucleotide sequences 37
hapmat: A specific codec for compression of a haplotype matrix 38
DomQual: a specific codec for compression of base quality scores 39

7. Random access, subsetting & pipeline integration 41

8. Tools for obtaining statistics and metadata 43

9. CPU scalability: synchronisation and thread management 45

10. Security 46

11. Genozip file format 47

12. Detailed results data 49

1. Genozip high level architecture

Figure S1 (same as Figure 1 in main text) - Genozip high-level architecture. The Genozip framework interprets and reads the input file(s) in the main thread (I/O thread) and divides them into vblocks, which are then segmented. Segmentation is followed by the compression step. Compressed vblocks are sent back to the I/O thread to create the .genozip output(s).

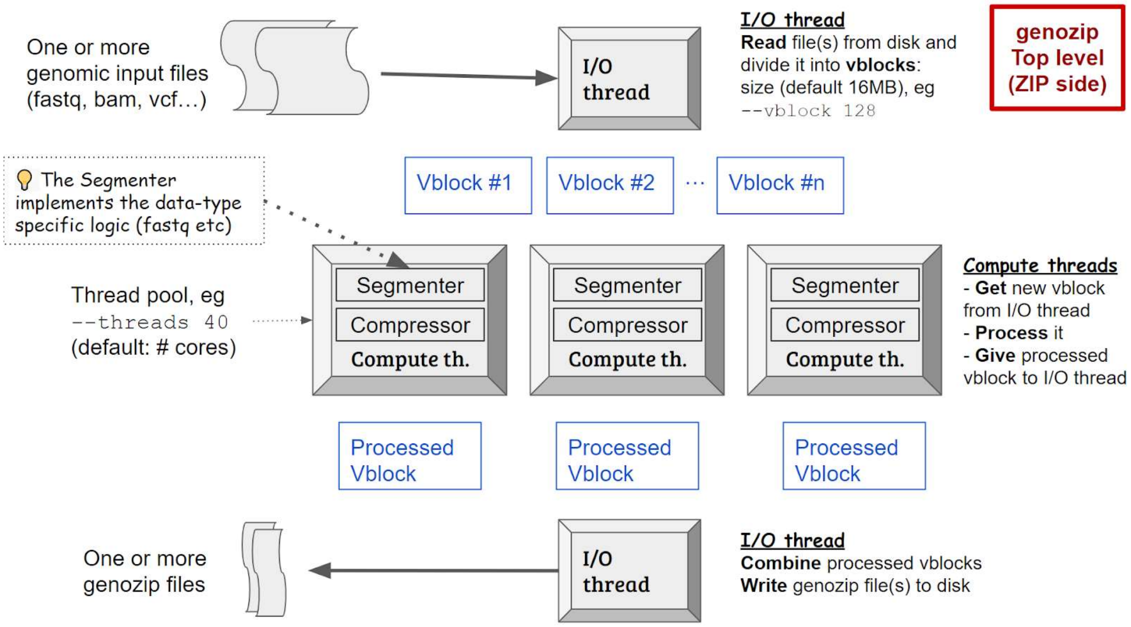

At a high level, Genozip is a C language program tested to run on Linux, MacOS and Windows (64bit). The same C program may be invoked by 4 command line commands: *genozip*, *genounzip*, *genocat*, and *genols*. The first two have major code components associated with them, described below. *genocat* essentially executes *genounzip* where the output goes to stdout and flags useful in pipeline analysis are made available. *genols* is lists the genozip files in a disk directory along with some metadata.

Here we will describe the ZIP side (i.e. the compression side). The PIZ side (decompression) follows a very similar architecture, where the inverse operations are carried out as expected.

The system was designed so that the logic related to compression of specific data elements is mostly coded in the ZIP side, while the PIZ side is as generic as possible. The reason for this is to allow the compressor to evolve, adding compression algorithms to an ever growing list of data elements, without needing to change the decompressor or the file format. There are a few exceptions to this that will be discussed.

When run, a Genozip process consists of one main thread (called the *I/O thread*) that is responsible for parsing the user's command line, reading and writing files to disk, as well as splitting the data stream into *vblocks* and spawning additional threads (called *compute threads*) to do the CPU-intensive compression or decompression work.

On the ZIP side, a *compute thread* consists of two main stages:

- First to run is the *Segmenter*: this parses the uncompressed file data (called *txt data*) first into individual logical lines (that are actual ASCII lines in all supported formats, with the exception of FASTQ where every 4 ASCII lines are counted as one logical line), and then parsing each line into its components, and finally applying a compression algorithm specific to the type of each component. The output of these component compression algorithms is stored in data structures called *contexts*. The Segmenter is where most of the logic related to specific data types (SAM, FASTQ etc) is located, while the rest of the Genozip code is not data-type specific.

- Second is the *Compressor*: it compresses each context using the appropriate codec that can be a *generic* or *specific* codec, as listed in the main text.

On the PIZ side, a *compute thread* does the inverse:

- First to run is the *Decompressor,* which decompresses the *sections* as read from disk to recreate the *contexts*

- Second to run is the *Reconstructor* that is data-type specific, querying the various contexts to losslessly reconstruct the original data. The instructions of which algorithms to use for reconstruction are part of the data themselves and not hard coded in the *Reconstructor* (with some exceptions) — i.e., the *Reconstructor* effectively pulls data from contexts and applies various algorithms as directed by instructions in the data. Upon completion, the *txt data* is reconstructed in the *vblock*, ready to hand back to the *I/O thread*.

Each *compute thread* receives a single *vblock* from the I/O thread, processes it, and hands it back to the *I/O thread* upon completion. *Compute threads* run in parallel and might complete out-of-order. The *I/O thread* receives back the processed *vblocks*, writes them to disk in the correct order, with each *context* within the *vblock* written as one or more *sections* in the final Genozip output file. Genozip utilises as many processor cores as it can, unless the user limits the number of threads with `--threads`. A substantial part of Genozip consists of thread synchronisation algorithms that are designed to ensure maximum CPU core scalability with minimum bottlenecks.

2. The Segmenter

General

This section expands and adds details to the segmenter description in the main paper.

A *segmenter* is a module that is specific to the file format being compressed. Genozip currently has eight segmenters, one each for SAM, VCF, FASTQ, FASTA, GVF, PHYLIP, 23andMe, and Generic. More segmenters can be added to the open source code by interested parties.

The *segmenter* is called by the framework with one line of *txt data* at the time, and the job of the *segmenter* is to segment this line into its individual data components, store these in *contexts* which will be described hereinafter, and declare how each context should be handled during the compression stage.

The segmentation process from the Segmenter's viewpoint

We now explain how a segmenter works and how the framework interacts with one by walking through a simple example of a single *txt line*, and explain in detail the logic related to it. As an example, we use the following VCF line (the VCF header line is provided here only for clarity) and the VCF segmenter.

```
CHROM POS ID    REF ALT QUAL FILTER INFO             FORMAT Smp1   Smp2
chr12 0   rs123 G   A   100  PASS   AC=1;AN=2;AF=0.5 GT:DP  1|1:37 1|0:32
```

The first step for the VCF segmenter is to break the *txt line* into the top-level data fields, which are separated by a tab character in the case of VCF. This is a trivial task using macros provided by the framework.

Next, the VCF segmenter needs to decide what to do with each data field. Broadly, it has six options:

1. The segmenter may place the data directly in its appropriate *context*. This is the simplest case, and indeed the most common one. In the case of the *txt line* above, the VCF segmenter applies this strategy to the CHROM, QUAL, FILTER fields—placing them in the CHROM, QUAL and FILTER *contexts* respectively.

   The Genozip framework, in turn, would add these *snips*, to a *dictionary* within each *context*, if they are not already in this *context's* dictionary, and would place an index to the *dictionary* entry in a data buffer for this *context* called the *b250 buffer.* This way the Genozip file stores each *snip* only once, and uses a numeric index to point to it

throughout the file.

2. The segmenter may <u>further segment a field into its subfields</u>, and then for each subfield recursively apply one of these six options. In this particular *txt line*, the VCF *segmenter* segments the INFO field to its subfields, with each INFO tag being considered a field. The structure of the INFO field, including the number of subfields the tag prefixes (eg “AC=”) is represented in a data structure called a *Container*. This *Container* is created for this INFO field, and placed as a *snip* in the INFO *context*. The *Container* contains only the description of each *item* which together the *record* that is the INFO field (not the values themselves), so that *txt lines* that have the same tag names in the same order in their INFO field will have the same INFO *snip,* and hence the same index placed in the *b250* buffer. The VCF segmenter will now need to consider the values of the AC, AN and AF fields (“1”, “2” and “0.5”) and recursively apply one of these six options to each of them.

   A *Container* can also contain an array—in our example, the entire set of samples in a VCF line goes into one *Container* that describes the tags (GT and DP in this case), as well as the number of *repeats* (= the number of samples; in this case, 2)—and is placed in the SAMPLES *context*. As before, the GT and DP values are not included in the *Container*, and the VCF segmenter needs to recursively choose one of these six options for each of them.

   The GT field is further segmented into its individual haplotype values as well as its phasing value (‘|’ in this case). The VCF segmenter encodes this as a *Container* which contains an array where *repeats*=ploidy, and each array entry has a single item—the haplotype. The phase value is treated as the separator between repeats, which is also defined in the *Container* data. This *Container*, once again not containing the haplotype values themselves, is placed in the GT *context*.

   Similarly, Optional fields in SAM are an array of records, and any specific Optional field that is an array (i.e. SAM type ‘B’) is itself stored as a *Container* that is the array. The INFO field in VCF and the ATTRS field in GVF are a *Container* containing a record, with the items being the tags, etc.

   All segmenters are required to have a single *Container* per *vblock* that goes into the TOPLEVEL *context*. This *Container* describes the entire *vblock*—it is an array with *repeats*=number of lines in the *vblock*, and the items describe the structure of a line as defined for this file format. In the case of our example *txt line:* the TOPLEVEL has 10 items (CHROM, POS, ID, REF+ALT, QUAL, FILTER, INFO, FORMAT, SAMPLES and EOL), where INFO and SAMPLES are themselves *Container*s as described above.

   This is a key feature in enabling the decompressor to be generic. Indeed, the decompressor need not have any built-in awareness of the details of each file format because the file format structure is encoded in the data itself, and a *vblock* may be reconstructed by traversing the data starting from the TOPLEVEL.

3. The segmenter may <u>exploit known correlation between fields</u> in order to improve the compression. In the case of the *txt line* above, the VCF segmenter employs this strategy in two occurrences:

    a. Since the REF and ALT field are highly correlated, they are stored together ("G A" in this *txt line* example) in a single context REF+ALT.

    b. In the case of AC, AN, AF, the segmenter checks whether AC equals AN*AF as expected with the common use of these tags in VCF files. If this is indeed the case, the AN and AF fields are stored normally in the AN and AF *contexts* while the AC is simply stored as the *snip* "SPECIAL AC". Since normally we would expect all AC values in a *vblock* to be AN*AF, the AC dictionary will contain in this case only a single entry "SPECIAL AC" and the *b250* data for each *vblock* will have an entry for each line in the *vblock* for which we have these INFO tags, but all values will be the same: the *dictionary* index of the "SPECIAL AC" snip. This will cause the *b250* data to compress to a trivial size in the compression step.

       Using SPECIAL AC will then require providing an extension to the uncompress side called a *special reconstructor* for AC. `genounzip`, when encountering the "SPECIAL AC" snip in the *b250* of the AC *context*, will call the AC *special reconstructor* that will implement the specific special reconstruction algorithm, in this case simply emitting the value of AN*AF.

       Typically, we have a handful of special reconstructors for each file format that represent opportunities to compress based on relationships between fields or even use of external information.

       Important to note, it is not an error if AN*AF is not equal to AC. It is not the role of Genozip to enforce correctness of field values and it is always tolerant if the value is not as expected. In this case, it will simply store the value of AC in the AC context instead of "SPECIAL AC".

       SPECIAL algorithms may be as simple as multiplying AN and AF, or may be as complex as needed. For example, we use the same mechanism to analyse the SEQ field (nucleotide sequence data) in SAM and FASTQ files against an external reference file.

       SPECIAL algorithms are a powerful tool for encoding any type of relationship between fields, and as such, may contribute significantly to the compression ratio. However, this power comes at a cost, namely that the reverse algorithm for retrieving the original value from the encoded value in combination with the value of the related fields, must be encoded as a *SPECIAL reconstructor* on the decompressor side, thereby adding data-type-specific code to the decompressor side, which we are attempting to minimize.

4. The segmenter may: use one of the genozip's framework built-in algorithms. In our *txt line* example, we have two occurrences of this strategy:

    a. The POS field uses the built-in *seg_pos* interface. This inspects the POS value compared to the previous line.If the absolute value of the difference is at most 32000, it stores the snip "DELTA (*this_pos - prev_pos)*" in the POS *context*. If it is more than 32000, it stores "LOOKUP" snip in the POS context's *dictionary / b250*, and the value of POS itself as a 32 bit unsigned integer in the POS context's *local* buffer. `genounzip`, when encountering a "LOOKUP" snip, reconstructs the value from the *local* buffer.

    b. The ID field uses the built *seg_id* interface. This attempts to split a *snip* into an alphabetical and a numeric part—"rs" and "123" in our example. The *snip* stored in the *context*'s *dictionary* would be "LOOKUP rs" and the unsigned integer value 123 will be stored in the *context*'s local buffer.

5. The segmenter may prepare the data for a *specific* codec. The segmenter may store the data of a field in any proprietary way, in preparation for consumption by a *specific* codec in the compression stage. In our example, the VCF segmenter stores the haplotype data in a haplotype matrix stored in this *vblock* data structure, which will be later compressed using the *hapmat* or *gtshark* codecs.

6. The segmenter may declare the field to be an *alias.* Sometimes it is beneficial to store more than one field in a single *context*. For example, the INFO/END tag (not in our *txt line* example) is normally an integer with a value between this line's and the next line's POS value. Therefore, we alias INFO/END with the POS context, which combined with the *seg_pos* interface normally creates a series of DELTA *snips* with delta values smaller than either POS or INFO/END would have on their own.

A full list of how each of the eight segmenters in Genozip handles each data field appears in the Table S4**.**

Pre-segmentation *Cloning* and post-segmentation *Merging*

When the *segmenter* for a particular *vblock* is complete, the framework merges each *context* created in this *vblock*, with the corresponding global *context* held in memory in an object called the *z_file*. This merge does not affect the *b250* and *local* data that remains private to the *vblock*, and is focused mostly on the *dictionary* data. Since segmentation of many *vblocks* happens in parallel in multiple threads, each thread adding *snips* to their private *dictionary* fragment, with the possibility of multiple parallel threads adding some identical snips to their respective dictionary fragments, careful merger of the dictionary fragments into the *z_file* global dictionary, as well as re-writing the indices in the *vblocks*' *b250* buffer is required.

When the segmentation of a new *vblock* begins, the global dictionary is *cloned* from the *z_file* to the local *context* in the new *vblock*. For efficiency, no memory is actually copied but rather a set of parameters is set to determine which dictionary entries within the *z_file* dictionary are available to each *vblock*. This synchronisation algorithm is written carefully so that merging of completed *vblocks* into the global dictionaries may occur at the same time as active segmenters in other threads are accessing the very same dictionaries, and the access by segmenting threads can be done without the use of synchronisation objects like mutexes, which would create a bottleneck and limit the scaling to a large number of cores.

Singleton detection

The framework also includes a singleton detector: if a *snip* appears for the first time in a particular *vblock*, and appears only once in this *vblock*, then it is placed in the context's *local* buffer instead of in the *dictionary* and *b250*. This way, we avoid bloating global dictionaries with singletons and keep singletons local to the context. This is important, since *vblock* memory is freed once the output data of this *vblock* is written to disk, while the global dictionaries remain in memory until compression of the entire file is complete.

Hash tables & the Snip Diversity Estimation Algorithm

When a *segmenter* calls the framework to enter a *snip* into a context, the framework first needs to lookup that *snip* a the dictionary to know whether it is new and should be added to the context's local dictionary fragment, a new index generated, and that new index added to the local *b250* data, or whether this *snip* already exists, in which case the existing dictionary index should be added to the local *b250*. To perform this lookup in O(1), each context also contains a hash table that allows rapid lookup. To achieve O(1), it is necessary for the target range of the hash function, and hence the initial size of the hash table, to be proportionate with the total number of distinct *snips* across the entire file for any particular *context*. This varies drastically between *contexts*, and indeed, it can be very large for contexts for which we expect millions of distinct snips (resulting in a hash table size of tens of MBs of RAM or more) , or very small (in case the file has only a handful of unique values of a particular field) for contexts like FORMAT in VCF.

The challenge we face, is to estimate, without reading the entire file, how many unique values exist in the entire file for each particular context. For this we have developed the *snip diversity estimation algorithm*:

In most *contexts*, many new snips appear early in the file, but as we progress in the file, since many of the snips were already observed before, less new snips are encountered. To estimate the expected total number of snips of a particular *context*, we analyse the data for this *context* for the first *vblock* in which this *context* is encountered. We look at the first derivative within this *vblock*:the density of new *snips* within a context—d(new_snips)/d(lines),

as well as the second derivative—d(density)/d(lines). We then estimate the total file size, which might not be known if the file is compressed (with gzip, bzip2, xz, bgzip, bam or bcf) or if it is piped from stdin. We use the values of the first and second derivative and the estimated file size to estimate the number of unique *snips* across the entire file. Performing a simple mathematical integral to reach the results yields a poor match to the real values, and hence we enhance this with several heuristics based on observations of real world data. The full algorithm can be found in the function `hash_get_estimated_entries()`.

The Snip format

Snips stored by a Segmenter in a context may be simply the text of the data field itself; indeed, this is most often the case. However, Genozip has a number of snip opcodes that allow storing the data in a more compressible format, where applicable. In this case, rather than storing the data string as the snip, we construct a snip starting with one of the opcodes in the table below (each being one byte), followed by the required parameters. The decompression side has built-in algorithms for reconstructing the data field's string based on the data in the snip, while the *b250* array consisting of these snips is expected to be less random and/or with a smaller dictionary, and hence compress better than if we were to insert the data itself.

Table S1: Snip opcodes. When a snip has one of these values as its first byte, it is reconstructed as prescribed in this table, rather than just copying the snip.

Name	Parameters	Reconstruction algorithm
LOOKUP	*prefix* (optional)	*prefix* followed by the next value from *local*
OTHER_LOOKUP	*other_context* *length* (optional)	The value from *local* of *other_context*. If local is of type LT_SEQUENCE, then use *length* characters.
PAIR_LOOKUP	-	Copy value in the matching row in the paired file (when compressing FASTQ with `--pair`)
CONTAINER	*structure*	Recursively reconstruct the values from the contexts listed in structure, and combine them as specified
SELF_DELTA	*delta*	Value on previous line + *delta* (*delta* may be negative)
OTHER_DELTA	*other_context* *delta*	Last value from *other_context* + *delta*
PAIR_DELTA	*delta*	Value of matching row in paired file + *delta*
SPECIAL	*algorithm* *params (optional)*	Reconstructor to use the requested *algorithm* with *params*

The *Context* data structure

Genozip achieves its flexibility relative to file formats, by compressing individual data components of files into *contexts*, which are based on a recursive data format called *snips* which allows arbitrarily complex component-specific logic. It is recursive in the sense that *snips* might themselves be containers containing other snips.

Each context contains three data *buffers*:

1. The *dictionary.* This buffer is generated as the txt file is segmented, containing a single entry for each snip that appeared so far in the file. When a *vblock* segmentation commences, the dictionaries of all contexts, as updated by previously completed vblocks, are cloned into this vblock as are accessed on a read-only basis. If new snips are discovered in this vblock that are not already in their respective dictionary, vblock-private dictionary fragments are created. When a vblock segmentation completes, these fragments are integrated back into the global dictionary. Care is taken to make sure the global dictionary contains exactly one entry per snip, even though multiple vblocks running in parallel might discover the same snip and add it to their respective dictionary fragments.

2. The *b250.* This buffer contains 32-bit indices into the dictionary of all the snips of this *context* in a particular *vblock*, in the order they will be read by the decompressor. If no *b250* buffer exists, the decompressor will take the data from *local* (see below), but if it does exist, it must contain exactly one entry for each related data component in the txt file. To improve compression of the b250 buffer, some 8-bit values (instead of 32 bit) are used in some cases: A. if the snip the most, 2nd most or 3rd most frequent snip (as measured in the first vblock in which this context is used) B. If the snip index is one higher than the index of the previous snip in this b250 (this will result in a highly compressible run in some cases) C. missing non-GT values in VCF samples.

3. The *local* buffer. This buffer contains data that is private to this *vblock* and is not in the dictionary. Some contexts use the *local* buffer to contain singleton snips that are expected to appear, as determined by a heuristic algorithm, only in a single *vblock*, while some contexts use the *local* buffer to store all the data, when this data is expected to be mostly private to this *vblock*, rather than using a dictionary. A context may utilise *local* to store either *snips* or alternatively simple data, such as integers or bitmaps.

This context based data structure is extremely flexible because it is independent of any particular genomic file format: when coding a segmenter for a particular file format we may pick and choose the most appropriate algorithms for each context, or develop new ones if needed, and *genozip* as a system may evolve fast in the future, by easily updating

algorithms for specific contexts. Indeed, *genozip* can also serve as a good testing platform for new algorithms that focus on specific data elements of genomic data, by allowing creating or modifying contexts these specific elements.

We illustrate this by describing four contexts: POS (as it appears in VCF, SAM and 23andMe), ID (as it appears VCF, 23andMe, GVF), XA tag in SAM and Compound Field. A full list of all algorithms follows.

As explained above, a snip is created by the Segmenter (compressor side) is usually reconstructed as-is by the Reconstructor (decompress side), unless it begins with one of the special opcodes, which may be followed by parameters.

The built-in *POS* algorithm

The POS algorithm is one of the built-in framework algorithms that segmenters may use. It is designed for numeric fields that contain a 32-bit unsigned value, and have the property that subsequent lines tend to have values that are quite near each other. This is a characteristic of the fields that are a coordinate within a specific chromosome, and hence the name. For example, this is the case for the POS field in VCF, the POS and PNEXT fields in SAM, the POS field in 23andMe. It also appears in various optional fields.

This is a good example of how a particular algorithm, in this case one designed to handle POS data, works with the three context buffers.

In this example, let's assume we have 3 lines in a particular vblock of a txt file (for example a VCF or SAM file), with POS values of 1000, 1500 and 10000000:

Table S2: SELF_DELTA example. Example the contents of the *b250* and *local* buffers of a POS *context*, after segmenting the values 1000, 1500, and 10000000

**Value**	***dictionary***	***b250***	***local***
1000	"LOOKUP"	0	1000
1500	"SELF_DELTA 500"	1	
1000000		0	1000000

As we can see, 1000 is the first POS value in this *vblock*. Since this is the first POS value, it will be stored as an unsigned 32bit integer in *local*, and the snip "LOOKUP" is added to the dictionary—telling the decompressor to lookup the value in local. Finally, the value 0, the index of the snip "LOOKUP" in the dictionary, is added to *b250*. 1500, the second POS value, is encoded as a delta vs. the previous line. Hence we add the snip "SELF_DELTA 500" to the dictionary, store the dictionary index of this snip, 1, in *b250*, and nothing in *local.* The third POS value, 1000000, is deemed to be too distant from the previous value 1500—beyond the defined threshold which is 32000—to be worthy of a delta. It is therefore stored as a "LOOKUP"

snip. We already have a “LOOKUP” in the dictionary, so we needn’t add another one: just place its index, 0, in our *b250* and the value, 1000000, in *local*.

The built-in *ID* algorithm

This is another build-in algorithm the framework provides segmenters.

Genomic data often contains IDs that are structured as a string containing a letter prefix, followed by a numeric suffix. Examples include the ID field in VCF, Dbxref attribute in GVF and EnstID identifiers that often appear in GVF attributes.

Table S3: ID example. Example the contents of the *b250* and *local* buffers of an ID *context*, after segmenting the values “rs999”, “strange_id” and “rs123”

**Value**	***dictionary***	***b250***	***local***
rs999	“LOOKUP rs”	0	999
strange_id	“strange_id”	1	
rs123		0	123

In this example, rs999 is the first ID value in this *vblock*. It is separated to its numeric component, 999, which is stored in local, and its letter component, rs, which is combined with LOOKUP to create the snip “LOOKUP rs”. This instructs the decompressor to output “rs” followed by the value looked up from *local*. Finally, the dictionary index of this snip, 0, is placed in *b250*.

The second ID value, strange_id, does not comply with our assumption regarding the format of IDs, namely being composed of letters followed by numeric characters. We therefore stored it as a simple snip “strange_id”, with the dictionary index of this snip, 1, placed in *b250*. This demonstrates the general approach of the various context algorithms: a specific algorithm is designed to optimise the compression based on assumed data format, but the algorithm can always handle data which is not compliant to the format, as long as the general file format rules (as defined in the file format specification—e.g., the VCF or SAM) are not violated.

The third ID value, rs123, is similarly decomposed with the index of the already existing “LOOKUP rs” snip, 0, placed in *b250* and the numeric value in *local*.

The built-in *Container* algorithm

A Segmenter may define *Container* snips.

We saw some examples of *Container* snips above. Here we take a closer look at how a *Container* snip is formed.

A *Container* snip is one that contains 0 or more *repeats* of a collection of items, collectively called a record:

- All elements of an array have the same structure—each is a record of items
- The items are defined by their *context*
- Each item within a record might be of a different type and its values go into a specific context.
- Each item may have prefix—the same prefix is used for this item in all records
- Each item and the entire record might have a one or two character separator. The same separator is used for all records.

Note that the *Container* snip only defines the structure of data; the values themselves of each item are stored in their respective contexts. These values may themselves be *Container* snips, enabling the ability of genozip to define data formats recursively.

Let's look at an example—the SA:Z optional tag in SAM:
`SA:Z:chr1,1000,+,151M,10,2;chr2,2000,-,151M,10,2`

This tag is defined in **REF** as as an array of records:
"SA:Z:(rname ,pos ,strand ,CIGAR ,mapQ ,NM ;)+"

In this case of SA, the *Container* snip will look like this:
(prefix="SA:Z:", repeats=2, (@RNAME,','), (@POS,','), (@STRAND,','), (CIGAR,','), (@MAPQ,','), (NM:i,';'))

This *Container snip* contains a prefix, and 2 repeats of 6 items each. The first 5 items have a ',' separator and the 6th has a ';' separator. The decompressor reconstructing this snip will reconstruct the data by querying these six contexts (@RNAME, @POS, @STRAND, CIGAR, @MAPQ and NM:i), twice for each, as well as insert the prefix separators in the appropriate places.

In this case, @RNAME, @POS, @STRAND and @MAPQ are contexts that are shared between the SA:Z, OA:Z and XA:Z tags in SAM. CIGAR is shared with the primary CIGAR field of SAM, an NM:i is shared with the NM:i tag in SAM.

The *Container snip* logic is extremely flexible in its ability to represent different types of data. The number of items in a *Container snip* as well as the number of repeats and the separators is in no way fixed; indeed, every individual *snip* can be defined as needed.

The built-in *Compound Field* algorithm

A segmenter may use this algorithm to decompose a string value into logical components, by breaking it at predefined separators. The number of subfields is variable, and each occurrence may have a different number of subfields.

This context algorithm is used for the QNAME field in SAM and the Description lines in FASTQ and FASTA.

The *Compound Field* is built on top of a *Segmented* snip: it creates *contexts* for each subfield, and results in a *Segmented* snip with one record and all subfields. The Container snip itself is stored in the main context, while each subfield is stored in its own subfield context.

Let's look at an example: a QNAME field in the first two lines of a SAM file:
`A00488:21621:1078`
`A00488:21766:1078`

When processing each one of these values, the *Compound Field* algorithm splits this string by the separator which is ':' (colon) in this case. Each component is then placed in its ordinal Q*NAME context, with * being 0 for the first component, 1 for the second etc (we used the numerals 0-9 followed by A-Z). It then places a *Container snip* in the QNAME context:

The first value `A00488:21621:1078` causes 4 contexts to update:
Q0NAME.dictionary ← 'A00488'   Q0NAME.b250 ←0 (index into the dictionary)
Q1NAME.dictionary ← '21621'   Q1NAME.b250 ←0
Q2NAME.dictionary ← '1078'   Q2NAME.b250 ←0
QNAME.dictionary ← CONTAINER*(repeats=1, (Q0NAME,':'), (Q1NAME,':'), (Q2NAME, ''))'*
QNAME.b250 ← 0 (index into the dictionary)

The second value `A00488:21766:1078` causes 4 contexts to update:
Q0NAME.b250 ← 0 (identical to previous line, index into an existing snip in the dictionary)
Q1NAME.dictionary ← 'SELF_DELTA 145'   Q1NAME.b250 ←1 (delta vs previous line)
Q2NAME.dictionary ← 'SELF_DELTA 0'   Q2NAME.b250 ←1 (delta vs previous line)
QNAME.b250 ← 0 (*Container snip* is identical to previous line, i.e., same index)

Note that in the common case where the entire file has QNAME data of the same format, we will have only one one item (one *Container snip*) in the QNAME context dictionary, and the entire QNAME b250 will be a run of 0's, compressing to a trivial size. Similarly, the other components also typically compress very well either because they are frequently identical between consecutive lines, or a small delta between consecutive lines.

The built-in *Special Snip* mechanism

In most contexts, the Segmenter (i.e. compression side) forms snips with one of the built-in algorithms, therefore, no code is required on the Reconstructor (i.e. decompress) side that is specific to this context. This gives us flexibility of evolving genozip by improving how we compress various contexts by coding the Segmenter only, allowing older genozip decompressors to still correctly reconstruct the new files.

However, in a few cases the desired algorithm is specific to the data component at hand, and cannot be generalised—as described above for the INFO/AC field in VCF. In these cases, we create a "*Special snip*" that redirects the Reconstructor to execute a special algorithm. This requires code in both the Segmenter and Reconstructor.

A *Special snip* contains the ID of the algorithm, and optionally parameters of the algorithm. Two C functions implementing the logic of any particular special snip must be provided: one for segmenting during, and the other for reconstructing during decompression, and the special snips must be declared in the header file of their data type (eg vcf.h, sam.h etc).

When a Special Snip algorithm is defined by a Segmenter, the Segmenter need not use it for all lines. Indeed, for any particular data component, the segmenter may choose to store a Special Snip for some lines and it may store data directly for other lines. This is commonly done when we *expect*, based on our knowledge that a value of a field may be expressible by a formula of data in other fields and/or external data. The segmenter will check for any particular line, whether our formula is indeed correct for the data of that line—and if it is, store the Special Snip providing the information needed by the Special Snip's reconstructor function to reconstruct the data—or whether our formula is not correct for this line, in which case the segmenter can just store the data as is.

Full list of contexts used, by file type

Table S4: List contexts by data (file) type. Each data type as a default set of contexts listed in this table. Additional contexts may be created to store optional fields (INFO and FORMAT fields in VCF, Optional fields in SAM/BAM etc).

Name	Snip	*local* use
**SAM**		
RNAME	As-is. Used for both RNAME and RNEXT fields.	-
QNAME	Container: Compound field	-
Q?NAME	Components of compound field:<br>**If** numeric<br>SELF_DELTA<br>**Else**<br>As-is	
FLAG	As-is	TEXT: Singleton snips
POS	POS algorithm as described above	UINT32: POS values if too distant from previous POS for delta
MAPQ	As-is	TEXT: Singleton snips
CIGAR	As-is	TEXT: Singleton snips
PNEXT	OTHER_DELTA POS *delta*	UINT32: PNEXT value if too distant from POS for delta
TLEN	**if** a non-zero value that is the negative of the previous line: "Δ -"<br>**Else if** tlen>0 and pnext_pos_delta>0 and seq_len>0: *SPECIAL (*TLEN, tlen - pnext_pos_delta - seq_len)<br>**Else**: As is	TEXT: Singleton snips
OPTIONAL	Container—one item per SAM tag	TEXT: Singleton snips
SQBITMAP	-	BITMAP: 0 if the base should be taken from the reference, 1 if it should be taken from NONREF (used for SEQ and E2:Z)
NONREF	-	TEXT: Based that differ from the reference<br>(used for SEQ and E2:Z)

GPOS	-	UINT32: Position within the reference<br>(used for SEQ and E2:Z)
STRAND	-	BITMAP: 1 if forward, 0 if reverse complement<br>(used for SEQ and E2:Z)
QUAL	-	TEXT: QUAL data *or* DOMQUAL: data<br>(used for QUAL and U2:Z)
QDOMRUNS	-	UINT8: Dom run lengths if DomQual algorithm is used<br>(used for QUAL and U2:Z)
SA:Z	Container—array of: (@RNAME, @POS, @STRAND, CIGAR, @MAPQ and NM:i)	TEXT: Singleton snips
OA:Z	Container—array of: (@RNAME, @POS @STRAND, CIGAR, @MAPQ and NM:i)	TEXT: Singleton snips
XA:Z	Container—array of: (@RNAME, @POS, @STRAND, CIGAR and NM:i)	TEXT: Singleton snips
@RNAME	As-is (a subfield of SA/OA/XA)	TEXT: Singleton snips
@POS	-	UINT32: numeric values (a subfield of SA/OA/XA)
@STRAND	As-is for SA,OA ; sign of POS for XA	TEXT: Singleton snips
@MAPQ	As-is (a subfield of SA/OA)	TEXT: Singleton snips
NM:i	As-is—shared between NM:i tag and NM values within SA,OA,XA	TEXT: Singleton snips
MD:Z	**If** seq_len implied by MD:Z is identical to seq_len implied by CIGAR:<br>*SPECIAL (MD*, value where final number is replaced with ‘*’). For example: `119C31` → `119C*`. In many cases there will be just a number that equals the seq_len which will be replaced ‘*’, thereby making a highly compressible *b250*.<br>**Else**: As-is	TEXT: Singleton snips

BD:Z<br>BI:Z	**If** the length is equal to seq_len:<br>The data is stored in *local* of BD_BI context, with each two bytes representing one byte from BD and one byte which is the delta between the byte from BD and the corresponding byte from BI.<br>The BD and BI Snips themselves are: *SPECIAL* (BD_BI)<br>**Else**: As-is	BI data (either delta vs BD or As-is)
AS:i	***If*** *seq_len >= value*<br>*SPECIAL (AS, seq_len - value)*<br>***Else***<br>As is	TEXT: Singleton snips
Numeric array tags *:B	Container—one item, repeats=array_len	TEXT: Singleton snips
All other SAM tags	As-is	TEXT: Singleton snips
EOL	As-is End of line—either ‘\n’ (Unix-style) or ‘\r\n’ (Windows-style)	TEXT: Singleton snips
**VCF**		
CHROM	As-is	-
POS	POS algorithm as described above. Used for both the POS field and the INFO/END tag	UINT32: POS values if too distant from previous POS for delta
ID	ID algorithm as described above	UINT32: numeric component of ID
REFALT	**If** `--reference` / `--REFERENCE` is used:<br>**If** REF = reference value, set REF to ‘-’<br>**If** ALT = reference value, set ALT to ‘-’<br>**If** ALT is a single base (i.e. SNP) that is the common ALT of REF (A↔G; C↔T), set ALT to ‘+’<br><br>**If** either ALT or REF are ‘+’ or ‘-’<br>*Special (REFALT, REF, ALT)*<br>**Else**<br>*As-is*	TEXT: Singleton snips
QUAL	As-is	TEXT: Singleton snips
FILTER	As-is	TEXT: Singleton snips

INFO	Prefix followed by Container:<br><br>Prefix is the INFO string including the tag names, ‘=’ and ‘;’ but excluding the values<br><br>Container contains an item (context) for each INFO tag which has a value (repeats=1)<br><br>Example: “AC=1;AN=2;MYFILTER”<br>“AC=;AN=;MYFILTER<br>*Container* (repeats=1, (AC,”), (AN,”))”	TEXT: Singleton snips
INFO/SVLEN	**If** a SVLEN is negative and equal to POS-END<br>*Special* (SVLEN)<br>**Else**<br>*As-is*	TEXT: Singleton snips
INFO/AC	**If** AC = AN * AF<br>*Special* (AC)<br>**Else**<br>*As-is*	TEXT: Singleton snips
INFO/END	Alias of POS	
All other INFO tags	As-is	TEXT: Singleton snips
FORMAT	As-is	TEXT: Singleton snips
FORMAT/GT	Container with one item, and repeats=ploidy, with item separator being the phase character (‘/’ or ‘|’).<br><br>The haplotype data is stored in *GT.local*, as a matrix of lines x haplotypes. The matrix padded as needed to the maximum ploidy in this vblock, or in case of missing samples or lines in the vblock without GT. This matrix is then compressed at the compression stage with a *specific* codec, either *hapmat* (**REF**) or *gtshark* (**REF)**	
FORMAT/DP	**If** equal to INFO/DP<br>*OTHER_DELTA* (INFO/DP, 0)<br>**Else**<br>As-is	-
FORMAT/ MIN_DP	*OTHER_DELTA* (FORMAT/DP, DP-MIN_DP)	-

FORMAT/GL	**If** largest probability value can be calculated from the other values:<br>Remove the largest probability value<br>**Else**<br>As-is	-
All other sample subfields	As-is	There is no *b250* or *local* sections for sample data; *b250* data is stored for all subfields together in a special *genotype* section as described in REF
EOL	As-is End of line—either '\n' (Unix-style) or '\r\n' (Windows-style)	TEXT: Singleton snips
**GVF**		
SEQID	As-is	-
SOURCE	As-is	TEXT: Singleton snips
TYPE	As-is	TEXT: Singleton snips
START	POS algorithm as described above.	UINT32: START values if too distant from previous START for delta
END	POS algorithm as described above, with *OTHER_DELTA* vs START	UINT32: END values if too distant from START for delta
SCORE	As-is	TEXT: Singleton snips
STRAND	As-is	TEXT: Singleton snips
PHASE	As-is	TEXT: Singleton snips
ATTRS	Same as VCF INFO	TEXT: Singleton snips
ATTRS/ID	POS algorithm	UINT32: ID values if too distant from START for delta
ATTRS/ Dbxref	ID algorithm	UINT32: numeric component of ID
ATTRS/Variant_effect	*Container* with repeats as appears in the value, and 4 items: V0arEff, V1arEff, V2arEff, ENSTid	TEXT: Singleton snips
ATTRS/sift_prediction	*Container* with repeats as appears in the value, and 4 items: S0iftPr, S1iftPr, S2iftPr, ENSTid	TEXT: Singleton snips

ATTRS/ polyphen_prediction	*Container* with repeats as appears in the value, and 4 items: P0olyPhp, P1olyPhp, P2olyPhp, ENSTid	TEXT: Singleton snips
ATTRS/ variant_peptide	*Container* with repeats as appears in the value, and 3 items: V0arPep, V1arPep, ENSTid	TEXT: Singleton snips
ENSTid	ID algorithm	UINT32: numeric component of ID
V?arEff<br>S?iftPr<br>P?olyPhp<br>V?arPep	As-is	TEXT: Singleton snips
ATTRS/Reference_seq	As-is (also used to store Variant_seq and ancestral_allele)	TEXT: Singleton snips
ATTRS/ Variant_seq	Alias of ATTRS/Reference_seq	
ATTRS/ancestral_allele	Alias of ATTRS/Reference_seq	
All other ATTRS tags	As-is	TEXT: Singleton snips
EOL	As-is End of line—either '\n' (Unix-style) or '\r\n' (Windows-style)	TEXT: Singleton snips
**23andMe**		
CHROM	As-is	-
POS	POS algorithm as described above.	UINT32: POS values if too distant from previous POS for delta
ID	ID algorithm	UINT32: numeric component of ID
GENOTYPE	-	TEXT: 2 characters per genotype
EOL	As-is End of line—either '\n' (Unix-style) or '\r\n' (Windows-style)	TEXT: Singleton snips
**FASTQ**		
CONTIG	As-is	-
DESC	Container: Compound field	TEXT: Singleton snips

D?ESC	Components of compound field:<br>**If** numeric<br>SELF_DELTA<br>**Else**<br>As-is	
E1L<br>E2L<br>E3L<br>E4L	As-is End of line for each one of the 4 txt lines that make up a FASTQ logical line	TEXT: Singleton snips
SQBITMAP	-	Same as in SAM
NONREF	-	Same as in SAM
GPOS	-	Same as in SAM
STRAND	-	Same as in SAM
QUAL	-	Same as in SAM
QDOMRUNS	-	Same as in SAM
**FASTA**		
CONTIG	As-is (first component of description)	-
DESC	Container: Compound field	TEXT: Singleton snips
D?ESC	Components of compound field:<br>**If** numeric<br>SELF_DELTA<br>**Else**<br>As-is	
LINEMETA	*Special snip* containing instructions on how to reconstruct a contig or part of a contig in this vblock	TEXT: Singleton snips
SEQ	-	TEXT: sequence
COMMENT	-	TEXT: comment lines
**GENERIC**		
DATA	-	All data
**PHYLIP**		
ID	-	SEQUENCE: ID data
SEQ	-	SEQUENCE: SEQ data
EOL	As-is End of line (Unix / Windows)	TEXT: Singleton snips

3. Optimisations

Genozip, by default, is strictly lossless. However, it also offers optimisations that modify the data—modifications that are designed to be harmless for typical downstream analysis but significantly improve the compression ratio. The user may activate all optimisations with `--optimise` (or `--optimize` or `-9`) or alternatively, only specific optimisations with their respective command line options listed in Tables S5.

Table S5: Optimisations. Options that can be used with `genozip` that modify the data to make it more compressible. `--optimise` (or `--optimize`) combines all these options

Command line	File types	Algorithm
`--optimize-sort`	VCF<br>GVF	INFO (VCF) and ATTRS (GVF): Within each line, tags are sorted alphabetically
`--optimize-PL`	VCF	PL: Phred values of over 60 are changed to 60
`--optimize-GP`	VCF	GP: Numbers are rounded to 2 significant digits
`--optimize-VQSLOD`	VCF	VQSLOD: Rounded to 2 significant digits
`--optimize-QUAL`	SAM<br>FASTQ	QUAL (SAM, FASTQ) and U2:Z (SAM): quality phred scores are binned, an similar to Illumina binning **REF**, but extended
`--optimize-ZM`	SAM	ZM:B: Negatives are changed to zero, and positives are rounded to the nearest 10
`--optimize-DESC`	FASTQ	Replaces the description line with '@filename:read_number'
`--optimize-Vf`	GVF	Variant_freq: Rounded to 2 significant digits

4. Compression against a reference and the Genozip Aligner

Overview

Genozip provides the ability to compress against a reference genome, in four cases:

a. FASTQ files
b. Unaligned SAM files
c. Aligned SAM files
d. VCF files (REF and ALT fields)

The reference is used in two distinct ways: in cases c and d, the file contains the position in the reference file, and we simply compare the file data to the reference data at the position provided. In cases a and b, the file does not contain positional information regarding the location of a particular read, and we use the Genozip Aligner to generate this position. In some cases where SAM files contain lines with and without POS information, we may compress the lines relying on the POS information where it is provided, and use the Genozip Aligner where it is not.

Most (if not all) aligners currently available have the objective of finding the true location an actual DNA fragment had in the original DNA molecule prior to the sequencing process. In contrast, the Genozip Aligner doesn't attempt to find the true location of a read, all we need to find is a location in the reference file that is significantly similar to the read so that we can use this similarity for better compression. This subtle difference in objective allows us to create an algorithm that is radically different from traditional aligners, trading off positional accuracy for speed.

The algorithm is divided into two:

1. Processing of a FASTA file into a reference file with `genozip --make-reference`. This step needs to be run only once, and the resulting reference file may be used to compress subsequent data files of the same species. The resulting reference file, distinguished by an extension `.ref.genozip`, contains mostly sections of two types REFERENCE and REF_HASH which shall be described below.

2. Compressing a data file against a reference file with either `genozip --reference` or `genozip --REFERENCE`. In the former the reference file needs to be provided to `genounzip` when decompressing, while in the latter the needed parts of the reference are stored as part of the compressed file, so the reference file is not needed for `genounzip`. This is particularly useful when binding together (i.e. compressing into a single `genozip` file) multiple files for delivering to a customer, as the cost in file size of storing the reference is amortised across multiple data files, and the customer doesn't need to worry about dealing with a reference file.

The REFERENCE data

When generating a reference file with `genozip --make-reference` *`fasta-file.fa`* a REFERENCE section is outputted for each *vblock* of FASTA data processed. The division of the fasta file into vblocks is constrained so that each *vblock* contains data from only a single contig—possibly the whole contig if it is short enough to fit in a *vblock*.

The REFERENCE data is simply a 2-bit representation of the FASTA data, where 'A' and 'a' are represented by 00, 'C' and 'c' by '10' (1) ; 'G' and 'g' by '01' (2) ; 'T' and 't' by '11' (3). Any other character contained in the FASTA data, including 'N', is represented by 00 as well. Every four characters are fit into a 8-bit byte, and the section is further compressed with *lzma*.

Note regarding bit notation: throughout Genozip, we store bits in bit arrays. These bit arrays are made out of 64 bit words, which is the native word size in most modern CPUs. When describing these bit arrays in this paper, we do so in two equivalent ways:

1. Little Endian: thinking of the bit array as a string of bits corresponding to a string of nucleotides—we write it e.g. 'ACG' as '001001'—enclosed in a single quote.
2. Big Endian: we can also think of the bit array as a binary number. Consistent with the normal way of describing numbers, we start with the most significant bit(without quotes, and prefixed with 0b) 0b100100 in this example, which is 36 in decimal.

We also store for each contig its GPOS (short for Global Position), a 32-bit unsigned integer that starts from 0 for the first contig, and is set for each subsequent contig to be higher than the (GPOS + length) of the previous contig.

The Genozip Aligner uses GPOS for describing the position of reads in the reference rather than (contig, pos).

We chose to store GPOS in an unsigned 32-bit integer, thereby limiting our Genozip Aligner to the first 4 Gbp of a reference. This is sufficient for single-species references commonly used today. For example, GRCh38 contains about 3.2 Gbp.

In the future, we might want to support references larger than 4 Gbp, in particular multi-species references that might be useful in metagenomics, which will require GPOS to be longer than 32 bits. Genozip already treats all position data (POS and GPOS) as 64-bit integers internally, so this could be relatively easily supported, but with the cost being achieving slightly worse compression ratios as GPOS data is also contained in `genozip` files of data files compressed using the Genozip Aligner.

The REF_HASH data

The second big chunk of data generated when generating a reference file with `genozip --make-reference` *`fasta-file.fa`* is REF_HASH data. This is a pyramid of ***num_layers*=**4 hash tables, of levels [0, 3], where each hash table contains $2^{28\text{-}\boldsymbol{layer}}$ entries. Each entry is a 32-bit unsigned integer, which will contain a particular GPOS, or remains at the initial value 0xffffffff if not used.

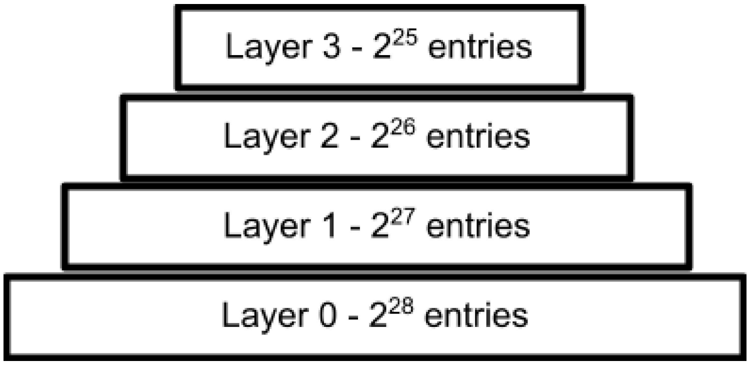

Figure S2: ref_hash tables. Layer 0 is attempted first, and if it is occupied, progressively higher layers are utilised

The ref_hash tables are created by a single traversal of the reference data described above, from ***gpos***=0 to the last GPOS value:

create_ref_hash:
    Initialise all ref_hash table entries to 0xffffffff
    Foreach locus ***gpos*** *in* ***reference*** which is 11 (i.e. ‘G’ or ‘g’) {
        If base at (***gpos***+1) is not also 11 {
            Let ***idx*** ← value of the next 28 bits (i.e. 14 bases) following the G
            Insert (***idx***, ***gpos***)
        }
    }

*Insert (**idx**, **gpos**)*:
    If (ref_hash[0][***idx***] == 0xffffffff) then ref_hash[***layer***][***idx***] ← ***gpos***
    Else If (ref_hash[1][***idx***] == 0xffffffff) then ref_hash[***layer***][***idx***] ← ***gpos***
    Else If (ref_hash[2][***idx***] == 0xffffffff) then ref_hash[***layer***][***idx***] ← ***gpos***
    Else If (ref_hash[3][***idx***] == 0xffffffff) then ref_hash[***layer***][***idx***] ← ***gpos***
    Else if (random chance of 25%)
        ref_hash[random(0 to 3)][***idx***] ← ***gpos***

Notes:

- The notation ref_hash[***layer***][***idx***] means the ref_hash table at layer ***layer***, at the index ***idx*** where only the needed least significant bits of idx are used (28 bits for level 0 down to 25 bits for level 3).

The resulting 4 ref_hash tables are compressed with *lzma* and written to the genozip reference file.

Compressing aligned SAM data

When compressing aligned SAM data (i.e. a SAM line for which we have the RNAME and POS), we use the ***reference*** data in the reference file, however we don't need the ***ref_hash*** tables and the GPOS data.

We process the data into the following data structures:

- SQ_BITMAP context: a bit array for which we have 1 bit for each base in the sequence which according to the line's CIGAR string consumes both Query and Reference as defined in the SAM spec **REF** page 8. The bit will be one if the decompressor should copy this base from the ***reference*** and 0 if it should get it from NONREF.

- NONREF context: a character array that stores all the bases that are different from the reference or that are not one of 'A','C','G','T'.

- REF_IS_SET: a bit array that contains one bit for each base (2 bits) in ***reference***. The bit is 1 if and only if the value of ***reference*** at this location will be needed for reconstructing the data during `genounzip`. It is used only in case of --REFERENCE (i.e. not --reference), to determine which parts of the reference should be written to the file.

The SAM segmenter, when segmenting a particular line within a *vblock* of SAM data, traverses the SEQ data according to the CIGAR data:

Foreach ***base*** in SEQ:
    Let ***cigar*** be the Op in CIGAR string covering ***base*** (as defined in **REF**):
        If ***cigar*** ∈ { 'M', '=', 'X' }
            If (***base*** == ***reference*****[***RNAME,POS(base)***]**)
                SQ_BITMAP ← 1
                REF_IS_SET**[***RNAME,POS(base)***]** ← 1
            Else
                SQ_BITMAP ← 0
                NONREF ← ***base***
    Else if ***cigar*** ∈ { 'I', 'S' }
            NONREF ← ***base***

Notes:

- *POS*(*base*) is the POS value of base calculated from the POS value in the line, and the CIGAR string, relative to the position of the base in the SEQ string
- Assignment to SQ_BITMAP and NONREF means adding one value at the end of the array

Compressing VCF data (REF and ALT fields) with a reference

When compressed without a reference, we store the REF and ALT data together (separated by a tab character) in the REFALT context. We do this as they are obviously correlated and hence storing them together results in better compression than storing them in separate contexts.

We the user specifies a ***reference*** (using `--reference` or `--REFERENCE`) we do the modify the string stored in the context in the following way:

If REF or ALT is the same base as in (CHROM,POS) in ***reference***, we store '-' instead of the base.
If ALT is the *common_snp*(REF), then we store '+' instead of the base.

Notes:

- *common_snp:* A↔G ; C↔T

- Since we don't force the user to use the same reference for compressing as used for generating the VCF, we can't assume the value of the REF field is the same as in ***reference***. However, we expect this to be the usual case. In this case, all the REF values will be replaced with '-'

- We also set REF_IS_SET exactly as described above for aligned SAM compression

The effect of this is to make the b250 data of the REFALT context a lot more compressible due to both the reduction of the dictionary size as well as the abundance of the "-\t+" word.

The significance of this algorithm on the overall VCF compression ratio depends on the significance of the REFALT contribution to the file's information content. In files that don't contain any samples or INFO data, the REFALT data tends to be a major contributor to the information content, while in files that are rich in INFO data and contain many samples with multiple subfields, the contribution of REFALT information to the file's information content will be minor.

Compression of FASTQ sequence data and SEQ fields in unaligned lines in SAM data

For a read sequence data (which we will refer as SEQ hereinafter) for which we don't have alignment information, as is the case in FASTQ and unaligned lines of a SAM file, we use the REF_HASH data to find the a ***gpos*** value which represents the beginning of a region of the ***reference*** that we choose to compress against. We attempt to find a ***reference*** region identical to the sequence at hand, or with a small amount difference.

When loading the ***reference*** data from the reference file, we store two copies of it in memory—one forward copy and one reverse complement copy.

Notes:

- We do not attempt to find the region with the absolutely smallest amount of difference, however this is very often the outcome nevertheless, in particular in the common case where there is only a single region in the genome with which the SEQ aligns reasonably well.

- We do not handle insertions and deletions (Indels), resulting in reads which contain Indels typically aligning well only to the longest sub-read segregated by Indels. However, since typically insertion and deletions appear only in a small percentage of the reads, this has minimal effect on the compression ratio.

- When comparing sequences to the ***reference***, we compare to both the forward and the reverse complement references. The ***gpos*** value stored is the lowest gpos of a base, which is the first base in case SEQ aligns to the forward reference, and the last base in case it aligns to the reverse complement.

- In SAM, It is possible that a *vblock* contains both aligned and unaligned reads. In this case, we will compress each read with the appropriate algorithm, resulting in the SQ_BITMAP and NONREF contexts containing data used to compress both types of lines.

- Since the Genozip Aligner algorithm does not handle Indels, it will not generate good compression ratios for reads created using long read sequencing technologies, such as PacBio SMRT or Oxford Nanopore, that are rich in erroneous Indels. These files will compress better if the data is first aligned into a SAM/BAM file using an appropriate aligner, and then compressed as an aligned SAM.

We process the data into the following data structures:

- SQ_BITMAP context: a bit array for which we have 1 bit for each base in the sequence at hand. The bit will be one if the decompressor should copy this base

from the ***reference*** and 0 if it should get it from NONREF. Note that unlike compressing aligned SAM reads described above, here we have no CIGAR.

- NONREF context: a character array that stores all the bases that are different from the reference or that are not one of 'A','C','G','T'.

- GPOS context: the context *local* data stores unsigned int GPOS values, one for each read. This is the lowest GPOS value of the sequence at hand as explained above.

- STRAND context: the context *local* data stores a bitmap, one bit for each read, which is 1 if this read is to be reconstructed against the forward reference and 0 if it is to be reconstructed against the reverse complement reference.

- REF_IS_SET: used exactly as described for aligned SAM compression.

align (SEQ):

Foreach ***base*** in SEQ:

If ***base*** is a 'G' and the preceding base is not a 'G':
score, gpos = *score_match* (SEQ, location of 'G' in SEQ, '*forward'*)
If ***score*** is the highest so far for SEQ
best_match ← ***score, gpos, '****forward'*

If ***base*** is a 'C' and the next base is not a 'C'"
score, gpos = *score_match* (SEQ, location of 'G' in SEQ, 'reverse')
If ***score*** is the highest so far for SEQ
best_match ← ***score, gpos, '****reverse-complement'*

*score_match (**seq**, 'G' location, **strand**)*:

idx ← numeric_value(14 nucleotides following the G)
gpos ← ref_hash[***idx***]
ref ← copy of the region of ***reference*** *or* ***reverse-complement-reference*** (determined by ***strand***) which is aligned to ***seq*** according to ***gpos***

Implementation details: at this point ***seq*** and ***ref*** are two bit arrays of identical length. Every two bits represent a nucleotide (0=A,1=C,2=G,3=T). The arrays are implemented as 64-bit words, so that each 64 bit word contains (up to) 32 nucleotides, starting at the first bit of the first word, and with the redundant bits of the last word set to zero.

score ← length (***seq***) - *count_1_bits* (***seq*** bitwise-XOR ***ref***)

Note: *count_1_bits* counts the number of bits that are '1' in a bit array

<u>Example</u>:

SEQ=”CACTCT**G***TTCGCAGCAGTCTG*CGCCCTTACACAAAATG”

Consider the 14 nucleotides following the G: for example:

“CACTCT**G***TTCGCAGCAGTCTG*CGCCCTTACACAAAATG”

Or, the 28 bits representing the same 14 nucleotides:

‘1111100110000110000111101101’ which is numerically 0b10110111100001100001100111111 (in binary) or 192,438,687 (in decimal).

gpos ← refhash[192,438,687] = 500000000 (example)

500000000 in this example is the coordinate in the whole-genome reference of the **G**

Consider the reference segment around this G, so that it is aligned to SEQ:

```
  SNP                        SNP      DEL
REF:”CATTCTGTTCGCAGCAGTCTGCGCCCTTTCACAAAGAT”
```

Using 64-bit words, bitwise-XOR the reference segment and SEQ

```
SEQ:’1000101110110111111001100001100001111011011001101010111100100010 000000001101’
REF:’1000111110110111111001100001100001111011011001101010111111100010 000000010011’
XOR:’0000010000000000000000000000000000000000000000000000000011000000 000000011111’
     WORD #1                                                          WORD #2
```

score = seq_len_in_bits - count_1_bits(XOR) = 74 - 8 = 66

Alternative contig names

When compressing a file using a reference, if a contig name that appears in the file does not appear in the reference, we attempt to search for it using alternative names:

- A number eg “22” is also searched with a “chr” prefix, “chr22”
- “M” and “chrM” are also searched as “chrMT”

The alternative contig names are searched and assigned during compression, and the mapping is stored in the genozip file in the `SEC_ALT_CHROMS` section.

Discussion

The algorithm scores candidate matches (alignments) by doing bitwise operations on entire 64-bit words containing 32 bases each. The entire scoring takes only a few CPU operations per 64-bit word, as little as 16 (depending on the CPU). This is the key component that makes the Genozip Aligner extremely fast.

Note that rather than counting the number of mismatching bases, we count the number of mismatching bits, and select the “match” (i.e. the reference locus) with the least mismatching bits. This is done with a single CPU instruction per 64-bit word on most modern CPUs (for example, `popcntq` on Intel CPUs). As a result, we don’t necessarily select the match with the least mismatching bases. However, in practice, the majority of reads will have exactly one locus which provides a good match which will be selected, and in the case where we have two or more loci that are all good matches (i.e., very low mismatching bases) and the one we select, with the lowest mismatching bits, is not the one with the lowest mismatching bases; this minute difference would be insignificant for compression purposes.

Sensitivity to Indels: since we compare whole bit arrays, we don’t handle Indels. This usually means that the match selected for a read that contains an Indel would be the one matching the largest sub-read, as segregated by Indels. For typical short-read data, the percentage of reads containing Indels is sufficiently small that this approach results in very good compression ratios. However, long read technologies available at this time often generate reads that are enriched in Indels that are not correct biologically but are rather artifact of limitations of these technologies. This algorithm does not work well with this type of long read data, and we advise users to first align the data using an appropriate aligner, and then compress the resulting SAM (or BAM) file, which would use the aligned SAM algorithm described earlier.

Computational complexity: Since the number of loci we test is proportional to the length of the read (we test all ‘G’ bases for forward matching and all ‘C’ bases for reverse complement matching), and each test compares the entire read to the candidate region in the reference;

the complexity of aligning a SAM or FASTQ file is $O(r^2n)$ with r being the read length and n being the number of reads.

Optimisation: if the command line option `--fast` is specified, we test every 5th base to see if it is a ‘G’ or ‘C’ rather than every base. On our test data, this resulted in a speed up of the alignment by about 4X at the cost of a 20% worse compression ratio.

5. Compression of FASTQ paired end read files

Genozip provides a command line option `--pair` that further optimises compression in case of paired-end FASTQ files, such as Illumina. When using this option, every two consecutive input files on the command line are assumed to be a pair.

This optimisation consists of:

1. For each component of the Description (i.e. the D?ESC contexts): if the value of the second paired file is identical to the first, store the snip LOOKUP_PAIR instead of the value. For most D?ESC contexts (all but one in Illumina files), the entire *b250* data would be a run of LOOKUP_PAIR compressing to a trivial size.

2. For GPOS in the second paired file: we store it as a PAIR_DELTA snip vs the GPOS of the first file.

3. For STRAND (in the second pair file): we store '1' if the second paired read direction (i.e. forward or reverse complement) is identical to that of the first paired read, and 0 otherwise. This is expected to result in long, highly-compressible runs.

6. Specific codecs

acgt: A *specific* codec for compression of nucleotide sequences

We observe that *lzma* compresses nucleotide sequence data well, however it is very slow. *acgt* is a new codec we present here, specifically for nucleotide sequences, which is about 25X faster on our test data at a cost of only 5% worse compression than *lzma*. We use *acgt* for compressing NONREF data in FASTQ as well as aligned and unaligned SAM, unless the user specifies `--optimize-SEQ` or `--optimize` in the command line, in which case we compress NONREF with *lzma*.

acgt compression is designed for nucleotide sequence data in which 'A', 'C', 'G' and 'T' characters make up the vast majority of the data, while other characters, such as 'N', are rare. The source data is expected to be textual ASCII data.

acgt splits the data into two streams, each which is outputted to a separate section in the genozip output file.

The first stream, CODEC_ACGT, is simply the sequence encoded in 2-bit as we do in REFERENCE explained above: 'A'/'a' are encoded as 0b00, 'C'/'c' as 0b01, 'G'/'g' as 0b10, 'T'/'t' as 0b11 and everything else as 0b00. The size of the data is ¼ of the original size since we're converting each byte into 2-bits. We then further compress the CODEC_ACGT with *lzma*.

The second stream, CODEC_XCGT, is used only if we have one or more characters that are not 'A', 'C', 'G' or 'T'. This is an array of bytes of the same length as the source data. We set it to 0 for every character that is 'A', 'C', 'G' or 'T' in the source data, 1 for every character that is 'a', 'c', 'g' or 't' (i.e. lowercase) and we leave other characters as they are in the source data for all other characters. We then compress CODEC_XCGT with *bz2*. Since the *bz2* algorithm contains run-length encoding, and since we expect the vast majority of characters to be 0, this compresses to a very small size in real world cases we tested.

hapmat: A *specific* codec for compression of a haplotype matrix

For FORMAT/GT data in VCF files, we use the algorithm described in REF to compress a matrix, who's lines represent variants, columns represent haplotypes (here, we loosely use the term *haplotype* to describe the column of a specific sample at a specific location in the GT value, even if the sample is not phased), and each entry in the matrix is a single character representing the allele. genozip supports alleles up to 99, where alleles 10 and above are rewritten as a single ASCII character. This algorithm has been now implemented as a codec:

- For each haplotype column, count the number of alternate alleles (allele 1 to 99)
- Sort the haplotype columns by the count alternate alleles
- Transpose the sorted matrix
- Compress the transposed matrix with bzip2

The results of this compression are stored in two contexts:
GT_HT.local stores the compressed matrix
GT_HT_INDEX.local stores the permutation index that describes how to un-sort the matrix back to its origin.

We note that there are better algorithms for compression of a haplotype matrix based on Positional Burrows Wheeler Transform, as described in (**REF**), and this might be an area for improvement in the future.

DomQual: a specific codec for compression of base quality scores

Compression of sequences of base quality scores, as they appear in FASTQ files and in the QUAL field of SAM files, are often a harder problem than that of nucleotide sequences, because there is no reference data to which we can compare base quality scores. Further, different sequencing technologies and even different versions or options selected within the same sequencing technology, generate quality scores with radically different patterns.

Here, we introduce a novel algorithm to address a specific pattern of quality scores that is very common. This pattern is defined by having a single quality score that dominates the sequence.

In Genozip, we decide for each particular *vblock* of FASTQ or SAM data whether to use *DomQual* by sampling the first 500 quality scores (i.e. characters) of the base quality data of each of the first 5 lines of the *vblock* (in FASTQ a *vblock* line means a 4 textual lines of the FASTQ file). If there is a single character that accounts for at least 50% of the number of characters sampled, then we use *DomQual* for the base quality data in this *vblock*, and set ***dom*** to the dominant character of the sample.

In real-world data we tested, *DomQual* will usually triggered in Illumina files with quality binning (**REF**) where the dominant character is usually 'F', Pac Bio CCS data where the dominant character is usually '~', and quality data that has been binned with the genozip option `--optimize-QUAL` or `--optimize`.

We consider the entire base quality data of the vblock as a single long sequence of quality scores. *DomQual* segments this sequence data into the *local* data of two contexts:

- QUAL context contains a copy of the sequence, with two changes:

1. All ***dom*** characters removed
2. For each remaining character (which by definition is a non-***dom***): if this character is NOT preceded in the source sequence by a *dom run* (which we hereby define as one or more consecutive ***dom*** characters), a byte with the value of 1 is inserted before this non-***dom*** in QUAL.

- QDOMRUNS context contains a length of each *dom run*, the sub-sequence of the source sequence containing one or more ***dom*** characters, preceding each non-***dom*** character, except those we marked with 1. These are represented by a single byte indicating the length (between 1 and 254). If the length of the *dom run* is more than 254, then we add 1 or more 0xff characters each, representing a length of 254. Example: 0xff 0xff 0x08 indicates a *dom run* of length 254 + 254 + 8 = 516.

The *local* data of both the QUAL and QDOMRUNS contexts is compressed with *lzma*.

Testing with Illumina binned quality data, we see that *bz2* achieves superior compression to *lzma*, and is faster than it. On our test data, the *DomQual* codec achieves 12% better compression than *bz2*, and is slower than *bz2* by a factor of 3.5X.

If the user specified the command line option `--fast`, we compress these contexts with *bz2* instead of *lzma*. On our test data, this resulted in a compression that is about 3% better and about 10% faster than *bz2* on the source quality sequence.

7. Random access, subsetting & pipeline integration

Genozip contains capabilities to allow genozip files to be directly integrated in analysis pipelines, as well as some internal subsetting capabilities.

`genozip` supports reading and writing txt files from a pipe, for example:

```
cat myfile.fq | genozip - --output myfile.fq.genozip
```

```
genocat myfile.fq.genozip | analysistool
```

Some analysis tools require random access to the txt file and hence cannot accept an input file on a pipe. In these cases, it would be necessary to genounzip the file first.

`genocat` is a tool for viewing the data within genozip file, and potentially subsetting it. The subsetting command line options are summarised in Table S6, more details are available by running `genocat --help`.

Random access (`--regions`) is implemented by a two global sections in the genozip file:

1. The `SEC_RANDOM_ACCESS` section is included in all genozip files of file formats on which `--regions` is supported. It contains an array for a record for each *vblock* (the list of contigs appearing in the *vblock)*, and for each contig, the first and last position within the contig appearing in the *vblock.* The contents of this section may be viewed using the `--show-index` command line option.

2. The `SEC_REF_RAND_ACC` section is included in reference files, and also in genozip files that are compressed with `--REFERENCE`. contains a similar array, but with a record for each `SEC_REFERENCE` section.The contents of this section may be viewed using the `--show-ref-index` command line option.

When using `genocat --regions`, genozip uses the information from these two sections to refrain from reading from disk *vblocks,* `SEC_DICT` sections and `SEC_REFERENCE` sections that contain no data from the requested regions.

Table S6: genocat options. A partial list of the options of `genocat` - those options that subset the file. See `genocat --help` for a full list of options.

**`genocat` option**	**File formats**	**Action**
`--downsample` `<rate>`	All	Include only one line (or read for FASTQ) per *rate* lines.
`--regions` <*region-list*>	VCF, SAM, FASTA, GVF, 23andMe, reference file	Include or exclude specific contigs and/or positions
`--samples` <*sample-list*>	VCF	Include or exclude specific samples
`--grep` <*string*>	FASTQ, FASTA	Show only reads (FASTQ) or contigs (FASTA) whose describe contains the *string*
`--drop-genotypes`	VCF	Exclude the FORMAT and samples columns
`--no-header`	All	Exclude the header lines
`--header-only`	All	Include only the header lines
`--header-one`	VCF, FASTA	In VCF, includes only the last of the header lines (with the field sane sample names).<br>In FASTA, includes only the first component of the description line (until the first space).
`--GT-only`	VCF	Exclude all sample subfields, except for GT
`--sequential`	FASTA	Output the sequence of each contig as a single line, removing any newlines
`--list-chroms`	VCF, SAM, FASTA, GVF, 23andMe, reference file	List the names of the chromosomes (contigs)

8. Tools for obtaining statistics and metadata

Genozip contains tools for obtaining additional information about the contents of files. These are summarized below. More details can be obtained by running `genozip --help -f`.

Table S7: Statistics and metadata options, provide deep insight into the data in the files being processes, as well as the execution flow of the Genozip algorithms

Option	Availability:<br>**Z** **genozip**<br>**U** **genounzip**<br>**C** **genocat**<br>**L** **genols**	Action
`--show-time`	ZUCL	Show profiling information of where execution time was spent
`--show-memory`	ZUCL	Show memory consumption information
`--show-stats`	Z	Show compression performance by context
`--SHOW-STATS`	Z	Show detailed context information
`--show-alleles`	Z	(VCF only) show alleles
`--show-dict`	ZUC	Show all dictionary fragments
`--show-one-dict` *<context>*	ZUC	Show dictionary fragments of *context*
`--list-chroms`	ZUC	List the names of the chromosomes (contigs)
`--show-gt-nodes`	Z	(VCF only) show the GT values matrix (transposed)
`--show-b250`	ZUC	Show contents of all *b250* sections (textual)
`--show-one-b250` *<context>*	ZU	Show contents of one *b250* section (textual)
`--dump-one-b250` *<context>*	ZUC	Dump the contents of a *b250* as it appears in the file (binary)
`--dump-one-local` *<context>*	ZUC	Dump the contents of a *local* as it appears in the file (binary)
`--show-headers`	ZUC	Show a subset of the contents of the genozip file section headers as they are read or written
`--show-index`	ZUC	Show the contents of the `SEC_RANDOM_ACCESS` section

Continued from previous page

**Option**	**Availability:**<br>**Z** `genozip`<br>**U** `genounzip`<br>**C** `genocat`<br>**L** `genols`	**Action**
`--show-reference`	ZUC	Show the ranges included the `SEC_REFERENCE` sections
`--show-ref-seq`	ZUC	Show the reference sequences
`--show-ref-index`	ZUC	Show the contents of the `SEC_REF_RAND_ACC` section
`--show-ref-hash`	ZUC	Show details of `SEC_REF_HASH` sections
`--show-ref-contigs`	ZUC	Show the details of the reference contigs
`--show-ref-alts`	ZUC	Show contents of `SEC_ALT_CHROMS` section
`--show-gheader`	ZUC	Show list of sections in this file, as it appears in the `SEC_GENOZIP_HEADER` section
`--show-vblocks`	ZUC	Show vblock headers as they are read / written
`--show-threads`	ZUC	Show thread dispatcher activity
`--show-hash`	Z	See the values of the parameters used for calculating the hash table size for each context
`--show-aliases`	ZUC	Show the `SEC_DICT_ID_ALIASES` section
`--debug-memory`	ZUCL	Show memory buffer allocations and destructions
`--debug-progress`	ZUC	See data related to the progress indicator
`--show-reference`	ZUC	Show details of the `SEC_REFERENCE` sections
`--show-is-set` *<contig>*	UC	Shows the contents of `SEC_REF_IS_SET` sections of *contig*
`--show-bgzf`	ZUC	Show details of BGZF blocks
`--show-containers`	UC	Show flow of container reconstruction
`--show-txt-contigs`	ZUC	Show contigs from the SAM/BAM header
`--show-mutex`	ZUCL	Show locks and unlocks of a particular mutex
`--show-digest`	ZUC	Show MD5 and Adler32 updates

source: the data in this table is based on the output of `genozip --help=dev`

9. CPU scalability: synchronisation and thread management

Genonzip threads are managed by a thread dispatcher. The dispatcher is used both by the main I/O thread loop as described in the architecture diagram, as well as for various secondary tasks throughout the code. The dispatcher dispatches raw *vblocks* to threads, collects processed *vblocks* upon thread completion, and updates the progress indicator.

The maximum number of concurrent threads is either set by the user with the `--threads` command line option, or is set to the available number of logical cores as retrieved from the operating system.

Actually utilizing a large number of cores is a challenge, as genozip contexted-oriented compression implies that the dictionary of each context is potentially grown by every *vblock* which contributes values to a dictionary that were not observed before. When multiple threads are running in parallel each attempting to update dictionaries, the synchronisation required (for example, blocking on a mutex while updating a dictionary), if implemented naively, would severely limit the number of threads that can actually run concurrently.

In addition to the dictionaries themselves, genozip also maintains a hash table per context, which allows an efficient search when a *compute thread* is searching for a dictionary index of a particular snip. These hash tables also evolve with each snip that is added to a dictionary.

To address this, genozip *context manager* maintains, for each context, a *z_context*. When a compute thread for a specific vblock starts, the *z_context* and associated hash tables are cloned into the vblock context. This cloning doesn't actually copy memory, but rather points *z_context* and hash tables, and includes various parameters to limit this compute thread's access to only the parts of the data that were available at the point in time of the cloning—effectively creating a read-only replica of the *z_context* at this point in time, but without the expensive operation of copying memory.

As the compute thread processes the vblock, it adds new discovered snips to its own private fragments, and its own hash tables.

After the vblock processing is complete, the context manager (running in the compute thread) merges this context's data back into the zfile data. Since multiple compute threads running in parallel may have added the same snip, these merges needed to be serialised and make sure that the snips added were not already added by a previous compute thread. For this synchronisation, we use mutexes in the most sparing way possible, and opting for carefully crafted sequences of CPU-atomic operations, therefore not blocking threads, in lieu of mutexes, wherever possible.

10. Security

DNA data is legally considered in many jurisdictions as “personally identifiable information” (PII) and as such is required to be secured.

Genozip provides built-in security that is easy to use.

When the `--password` is used, the genozip file is encrypted with the standard AES encryption, using the a 256-bit encryption key is generated for each section, derived from the password, the vblock number (*vb_i*) and the section type.

For padding the last block of each section to the 16-byte AES block size, a secure padding derived from the MD5 hash of the last 100 bytes of the section.

Accessing this file using `genounzip` or `genocat` is made possible only if the same password is provided.

In addition, using `genozip` with the `--md5` or `--test` options calculates the MD5 signature of the original txt file(s). Then, when using `genounzip` with `--md5`, the MD5 signature of the actual output file is compared to the MD5 stored in the genozip file, to ensure the file was not tampered with intentionally or accidentally.

Note that the MD5 calculated is that of the underlying textual file. For example, when compressing a .sam.gz or .bam file, the MD5 will be that of the underlying .sam file.

11. Genozip file format

The genozip file consists of *sections*, where each section is of a particular *section type* and consists of a *header* and a *body*. The header is a structure determined by the section type, while the body contains the actual data.

All numeric data is stored in Big Endian.

Genozip supports binding multiple files together into a single genozip file. We will refer to the compressed data of these each txt files as a *component* of the genozip file.

First to appear in the genozip file, are sections related to the *component* data. Each component consists of a `SEC_TXT_HEADER` section followed by 1 or more *vblocks*. Each vblock consists of a `SEC_VB_HEADER` section and all the contexts of this vblock which include any number of `SEC_B250` and `SEC_LOCAL` sections. In the case of VCF, these may also include `SEC_VCF_GT_DATA, SEC_VCF_PHASE_DATA, SEC_VCF_HT_DATA` and `SEC_VCF_HT_GTSHARK` sections.

Following the components, we have *global sections* that apply to the entire file.

As the last section of the file, we have the `SEC_GENOZIP_HEADER` section. This contains the header, a body which is the list of sections in this genozip file and their offsets, and, unlike other sections, this section also has a *footer* which appears at the very end of the file, and contains the offset of the beginning of the section.

When a genozip file is read (for example during `genounzip`), the footer is consulted first for the the offset of the `SEC_GENOZIP_HEADER` section, then the `SEC_GENOZIP_HEADER` is read, and then the required sections from the rest of the file, based on the section offsets that are in the body of the `SEC_GENOZIP_HEADER` section.

Below is an example of the sections of a VCF file. This is a very small VCF file containing 3494 lines of the VCF header data, followed by 6 data lines, each with just a handful of INFO tags, one sample and a few sample subfields. The list below is taken from the output of running genozip with the `--show-gheader` command line option.

Real-world genozip files can often contain tens of thousands of sections.

The full list of section types and format of the header of each appear in
`https://github.com/divonlan/genozip/blob/master/sections.h`

The first section is the *component* header, whose body is the VCF txt file header. This small genozip file contains only one component, but genozip supports multiple components.

```
 0. SEC_TXT_HEADER                          vb_i=0 offset=0 size=22277
```

First (`vb_i=1`) *vblock* header. This is a small VCF file that contains only one *vblock*

```
 1. SEC_VB_HEADER                           vb_i=1 offset=22277 size=93
 2. SEC_B250                 CHROM          vb_i=1 offset=22370 size=46
 3. SEC_B250                 POS            vb_i=1 offset=22416 size=46
 4. SEC_B250                 ID             vb_i=1 offset=22462 size=46
 5. SEC_B250                 REF+ALT        vb_i=1 offset=22508 size=46
 6. SEC_B250                 QUAL           vb_i=1 offset=22554 size=46
 7. SEC_B250                 FILTER         vb_i=1 offset=22600 size=46
 8. SEC_B250                 INFO           vb_i=1 offset=22646 size=46
 9. SEC_B250                 FORMAT         vb_i=1 offset=22692 size=46
10. SEC_B250                 AF             vb_i=1 offset=22738 size=41
11. SEC_B250                 AN             vb_i=1 offset=22779 size=41
12. SEC_LOCAL                AC             vb_i=1 offset=22820 size=41
13. SEC_VCF_GT_DATA                         vb_i=1 offset=22861 size=40
14. SEC_VCF_HT_DATA                         vb_i=1 offset=22901 size=40
```

This is the global area of the file. It starts with dictionary fragment sections—each *vblock* may or may not contribute a dictionary fragment to each context.

```
15. SEC_DICT                 CHROM          vb_i=0 offset=22941 size=999
16. SEC_DICT                 POS            vb_i=1 offset=23940 size=43
17. SEC_DICT                 ID             vb_i=1 offset=23983 size=42
18. SEC_DICT                 REF+ALT        vb_i=1 offset=24025 size=44
19. SEC_DICT                 QUAL           vb_i=1 offset=24069 size=42
20. SEC_DICT                 FILTER         vb_i=1 offset=24111 size=47
21. SEC_DICT                 INFO           vb_i=1 offset=24158 size=133
22. SEC_DICT                 FORMAT         vb_i=1 offset=24291 size=50
23. SEC_DICT                 AF             vb_i=1 offset=24341 size=42
24. SEC_DICT                 AN             vb_i=1 offset=24383 size=42
25. SEC_DICT                 AC             vb_i=1 offset=24425 size=45
26. SEC_DICT                 DP             vb_i=1 offset=24470 size=42
27. SEC_DICT                 RGQ            vb_i=1 offset=24512 size=42
```

This file was compressed with `--REFERENCE` and therefore contains the relevant parts of the reference data. First, in the list of all contigs in the reference file, followed by the reference data itself.

```
28. SEC_REF_CONTIGS                         vb_i=0 offset=24554 size=2665
29. SEC_REFERENCE                           vb_i=1 offset=27219 size=60
```

A list of context aliases

```
30. SEC_DICT_ID_ALIASES                     vb_i=0 offset=27279 size=44
```

These are an index describing the contigs and their position range within each *vblock* allowing for efficient random access, such as when subsetting with `genocat --regions`

```
31. SEC_RANDOM_ACCESS                       vb_i=0 offset=27323 size=52
32. SEC_REF_RAND_ACC                        vb_i=0 offset=27375 size=52
```

The genozip header

```
33. SEC_GENOZIP_HEADER                      vb_i=0 offset=27427 size=758
```

12. Detailed results data

Compressing against raw files

We evaluated the performance of Genozip by comparing it to several widely adopted genomic data compression tools using a set of standard benchmarking files provided by the National Institute of Standards and Technology's Genome in a Bottle (GIAB) project (Table S8).

Variant Call Format (VCF)
To benchmark Genozip's VCF compression performance, we compressed the GIAB v3.3.2 NA12878 single-sample VCF and compared the results against several other popular compression tools – gzip (Meyering n.d.), pigz (Adler 2014), BCF compression implemented in BCFtools (Li 2011), and bzip2 (Seward 1996).

Sequence Alignment Map (SAM)
To benchmark Genozip's SAM compression performance, we used the 30X downsampled BAM file from GIAB that was converted to SAM format using samtools v1.9 (Li et al. 2009). Genozip performance was compared to CRAM compression, both with and without sequencing quality binning (8 bins), obtained using Scramble v1.14.11 (Bonfield 2014), and also BAM compression (implemented in samtools v1.9; (Li et al. 2009)) and pigz (Adler 2014). For the reference-based compression methods (i.e. CRAM and genozip --reference) we used a slightly modified version of human reference GRCh37 that was created using the steps described by Luca Santuari (https://github.com/GooglingTheCancerGenome/sv-callers/wiki/Building-the-b37-human-decoy-reference-genome).

FASTQ
FASTQ is a widely used text format that stores sequence data and the corresponding qualities for each nucleotide. We used Bazam (Sadedin and Oshlack 2019) to generate paired-end FASTQ files from the same BAM file that was used for the VCF benchmark. We compared Genozip against several widely used file compression methods – i.e. gzip, pigz, unaligned BAM, and unaligned CRAM – as well as alignment-based methods that take advantage of sequence similarity to reduce data redundancy (providing similar comparisons to the tools tested in the SAM benchmarks).

The tests were conducted on a Linux machine with 56 cores.

In Table 2 and Figure 3 we describe the compression ratio achieved by Genozip vs other tools, as well as wall time observed.

Table S8: Benchmark files. Uncompressed files used for benchmarking compression of raw (i.e. uncompressed) files against other common tools.

File type	File size	Source
VCF	128 MB	ftp://ftp-trace.ncbi.nlm.nih.gov/ReferenceSamples/giab/release/NA12878_HG001/latest/GRCh37/
SAM	147 GB	ftp://ftp-trace.ncbi.nlm.nih.gov/ReferenceSamples/giab/data/NA12878/NIST_NA12878_HG001_HiSeq_300x/
FASTQ	2 x 112 GB	Derived from SAM file using Bazam (Sadedin and Oshlack 2019)

Table S9: Raw-file benchmark results. Results of compression of uncompressed genomic files with genozip and other commonly used tools for each file format.

Tool		Compression	Ratio	Compress time	Decompress t.
	**VCF**	1,807,059,769			
	.vcf				
pigz	.vcf.gz	113,699,782	15.9	1.9 sec	3.1 sec
bcftools	.bcf	153,988,419	11.7	23.82 sec	21.02 sec
bzip2	.vcf.bz2	71,358,351	25.3	260.05 sec	43.37 sec
genozip	.vcf.genozip	53,819,306	33.6	7.1 sec	6.53 sec
	**SAM**	510,942,582,641			
pigz	.sam.gz	148,212,447,723	3.4	00:12:40.3	00:34:17.4
samtools	.bam	157,455,536,282	3.2	00:23:16.7	00:29:48.5
scramble -9	.cram	109,288,249,883	4.7	00:27:58.4	00:17:34.4
genozip -e	.sam.genozip	88,329,235,177	5.8	00:33:41.1	00:27:55.3
Optimized cram: scramble -9B	.cram (-B)	85,327,476,246	6.0	00:48:56.1	00:19:10.4
Optimized genozip -9	.sam.genozip (-9)	67,589,616,986	7.6	00:30:51.1	00:20:38.0
	**FASTQ**	238,958,297,328			
pigz	.fq.gz	57,228,032,622	4.2	00:14:34.5	00:34:17.4
bwa mem | samtools sort | scramble -9	.cram	44,248,758,235	5.4	03:42:54.0	00:48:24.7
genozip -e	.fq.genozip	35,098,350,704	6.8	00:16:40.1	00:08:31.7
genozip -9e	.fq..genozip (-9)	12,837,612,728	18.6	00:08:52.3	00:05:26.4

Compressing against already-compressed files

Table S10: Genozip on already compressed files - Files used

File type	File size	Source
.fastq.gz	3.6 GB (R1+R2)	ftp://ftp-trace.ncbi.nlm.nih.gov/giab/ftp/data/NA12878/Garvan_NA12878_HG001_HiSeq_Exome/NIST7035_TAAGGCGA_L001_R1_001.fastq.gz ftp://ftp-trace.ncbi.nlm.nih.gov/giab/ftp/data/NA12878/Garvan_NA12878_HG001_HiSeq_Exome/NIST7035_TAAGGCGA_L001_R2_001.fastq.gz
.bam	147 GB	ftp://ftp-trace.ncbi.nlm.nih.gov/ReferenceSamples/giab/data/NA12878/NIST_NA12878_HG001_HiSeq_300x/RMNISTHS_30xdownsample.bam
.cram (lossless)	102 GB	Generated from the BAM file with: `scramble -9`
.cram (binned)	79.5 GB	Generated from the BAM file with: `scramble -9 -B`
.vcf.gz	128 MB	ftp://ftp-trace.ncbi.nlm.nih.gov/ReferenceSamples/giab/release/NA12878_HG001/latest/GRCh37/HG001_GRCh37_GIAB_highconf_CG-IllFB-IllGATKHC-Ion-10X-SOLID_CHROM1-X_v.3.3.2_highconf_PGandRTGphasetransfer.vcf.gz

Table S11: Genozip on already compressed files. Results of compression with Genozip of already-compressed files in formats in common use in research and medical settings. These results are also reflected in Figure 2 in the main text.

Source file		Genozip command `--optimise` added for the Optimised test	Genozip lossless		Genozip optimised	
**File**	**File size**		**Size**	**Factor**	**Size**	**Factor**
.fastq.gz	3.60 GB	`genozip --pair $file-R1 $file-R2 -e $ref-file`	1.24 GB	2.9 X	0.63 GB	5.7 X
.bam	147 GB	`genozip $file -e $ref-file`	82.3 GB	1.8 X	62.9 GB	2.3 X
.cram (lossless)	101 GB	`genozip $file -e $ref-file`	82.9 GB	1.2 X	63.6 GB	1.6 X
.cram (binned)	79.5 GB	`genozip $file -e $ref-file`	63.6 GB	1.2 X	63.6 GB	1.2 X
.vcf.gz	128 MB	`genozip $file -e $ref-file`	51 MB	2.5 X	50 MB	2.6 X

Compressing BAM vs compressing CRAM

In this test, we compared several aspects of Genozip's performance - compression ratio, time, memory and CPU usage, of compressing a CRAM file vs compressing the same data in BAM format. The tests were run on the same machine as the previous tests - one with 56 cores and over 700GB of RAM.

The CRAM file used was a 14GB file downloaded from ftp://ftp.sra.ebi.ac.uk/vol1/run/ERR324/ERR3241754/HG00731.final.cram and the BAM file used was a 37GB GB file generated from this CRAM file with `samtools view`. The results are in Table S12.

Genozip compresses CRAM files by using `samtools view` to first convert CRAM to SAM. In the decompression step, we piped `genounzip --stdout` into `samtools view -OCRAM` to recreate the CRAM file. The wallclock time in Table S12 represents the combined operation Genozip and `samtools`, while the CPU time and memory reflect only Genozip's, and not samtools', resource consumption.

In contrast, Genozip compresses and decompresses BAM files natively, without relying on `samtools` or `htslib`. Consequently, Genozip is free to scale to a much larger number of CPUs and complete the processing faster. The higher memory consumption in the BAM case (Table S12) is a reflection of Genozip's ability to scale to a larger number of CPU cores, and hence threads, in this case. The higher CPU time is mostly due to Genozip also decompressing the BAM BGZF compression (in `genozip`) and recreating BAM in compressed BGZF format (in `genounzip`) which it does not do in the case of CRAM, because the plain SAM data is piped in from or piped out to `samtools`.

Table S12: Compressing CRAM vs compressing BAM. Results showing Genozip's performance when compressing CRAM and BAM files containing identical data. With BAM, Genozip can scale to a larger number of CPUs.

	**CRAM - compress**	**BAM - compress**	**CRAM - decompress**	**BAM - decompress**
**Orig file size**	14482707829	38828072041		
**Compressed size**	13190514012	13530076092		
**Ratio**	1.1 X	2.9 X		
**Wall clock time**	14m 9s	7m 57s	13m 40s	5m 52s
**CPU time**	19017 sec	23211	8076 sec	18188
**Max memory**	14.5 GB	20.8 GB	10.8 GB	12.4 GB
**CPUs utilized**	22.4	48.7	9.8	51.6
